# Opening the Window of the Mind: Do Large Language Models Possess Human-Like Visual Attention?

**DOI:** 10.3390/jintelligence14100234

**Published:** 2026-10-01

**Authors:** Houji Jin, Ganyu Gui, Ziyi Wang, Zhicheng Cai, Zhijie Tang, Jiayi Zhang, Jian Wang, Huanghao Feng

**Affiliations:** 1 School of Computer Science and Engineering, Suzhou University of Technology, Changshu 215500, China; 2 Department of Language Science and Technology, The Hong Kong Polytechnic University, Hong Kong SAR, China; 3 School of Teacher Education, Suzhou University of Technology, Changshu 215500, China

**Keywords:** spatial language, spatial-layout recovery, lexical cues, construct validity, behavioral evaluation of language models, large language models

## Abstract

When people read a spatial description, their eyes move through empty space as if the described scene were present, a signature of imagery-driven attention documented by Spivey and Geng. Language models have no eyes, but they are routinely credited with spatial understanding on the basis of verbal answers, and such answers are ambiguous: a model can name the right direction by echoing a directional word the passage happens to contain. We ask whether responses to spatial descriptions are governed by the configuration described or by the vocabulary used to describe it. Adapting the Spivey and Geng materials, we hold each scene’s layout fixed and vary its linguistic support across four versions: one naming the direction, one conveying only the configuration, one reordering the same sentences, and one inserting a directional word that contradicts the layout. Five models from the Qwen and GLM families were probed with a forced-choice direction report, an unconstrained narration, a two-dimensional reconstruction, and a plain relational question, together with control conditions that vary the position and register of the inserted word, shuffle the object list in the prompt, remove the imagery framing, and offer an explicit indeterminate option. Analysis treats model-by-scene cells as units, with cluster-bootstrap intervals and mixed-effects models, across 14,900 responses. Passages that convey a layout without naming a direction are answered near ceiling, reconstructed and narrated in the correct object order, and unaffected by reordering, by shuffling the prompt’s object list, or by removing the imagery framing: directional vocabulary is not what these responses track. Three apparent limits on that competence proved to be artifacts of measurement. A misleading word is resisted on three quarters of trials when it precedes the layout and on none when it follows it, so the manipulation indexes recency rather than representational strength; a downward bias on axis-free scenes disappears once declining is permitted; and narrations judged unfaithful by direction coding are faithful when scored by object order. Layout recovery is robust, and spatial evaluations that vary a single response format risk measuring their instrument.

## 1. Introduction

In cognitive psychology, attention is regarded as a fundamental and pervasive phenomenon that is closely related to human cognitive ability (Petersen & Posner, 2012; Posner & Petersen, 1990). In a complex perceptual world, the intrinsic properties of surrounding objects modulate their salience so that some are far more likely than others to draw the eyes and hold the focus of gaze. A striking demonstration of how tightly language and this attentional machinery are coupled comes from mental imagery. When people listen to or read a description of a spatial scene, their eyes tend to move through empty space as if the described objects were actually present, tracing the layout of a scene that exists only in the mind (Altmann, 2004; Richardson & Spivey, 2000; Spivey & Geng, 2001). This imagery-driven gaze suggests that comprehension of spatial language is supported by an internal spatial model that is grounded in the same oculomotor system used for real perception (Franklin & Tversky, 1990; Kosslyn, 1994).

In recent years, generative large language models (LLMs) have made remarkable progress in natural language processing, producing fluent and semantically coherent text and achieving impressive results across a wide range of tasks (Vaswani et al., 2017). Because the core computation of these models is itself called an attention mechanism, it is tempting to ask whether they process spatial language in a manner that echoes the imagery-driven attention observed in humans. Posing that question carefully requires separating two things that are easily conflated. What can be measured in a text-only model is the content of its outputs: the direction it names, the object orderings it produces, the coordinates it assigns. What cannot be measured is whether any internal state resembles a percept or a spatial image. The present study therefore asks the tractable version of the question. It asks whether a model’s responses track the spatial configuration described in a passage, or merely the directional words that the passage happens to contain. Answering even this narrower question is complicated by an obvious confound: directional words such as up and down are abundant in text, so a model can often report the “correct” direction simply by echoing the words it has just read, without recovering any layout at all (Marcus & Davis, 2020).

To separate layout-driven responding from lexical association, we adapt the paradigm of Spivey and Geng (2001), whose stimuli describe scenes that unfold along a single dominant axis without ever naming the direction of gaze. We borrow their materials and their logic of holding a layout constant while varying its linguistic support; we do not treat our verbal measures as commensurable with their oculomotor ones. We reproduce their spatial families, upward, downward, leftward, and rightward scenes, extend each with a novel counterpart to guard against verbatim memorization of the original texts, and add non-directional control scenes. Every directional scene is then rewritten into four cue versions that hold the described layout constant while varying the linguistic support for it: an explicit version that states the direction outright, a de-directionalized implicit version that conveys only spatial layout, a scrambled version that preserves the same sentences but destroys their order, and a counter-cue version in which an inserted directional word points against the true layout. This manipulation turns a single question into a set of dissociations. If a model relies on surface lexical cues, its behavior should collapse once directional words are removed; if its responses are driven by the described configuration, removing the words should matter little, whereas contradicting the layout with a misleading word should reveal how firmly that configuration governs the response. That last expectation proved to be the naive one, and tracing why is a substantial part of what follows.

We probe five contemporary LLMs from the Qwen and GLM families, accessed through an application programming interface (API), with three complementary tasks: a forced-choice direction report, a free-form narration, and a two-dimensional reconstruction of the objects in the scene, collecting 6700 responses. These tasks let us examine not only whether a model reports the expected direction, but whether it preserves the relative ordering of the objects in a scene, how it behaves when word and layout disagree, and what its unconstrained narration looks like when no answer format is imposed. Additional control conditions decompose the counter-cue effect into insertion position, wording register, directional congruence, and non-spatial lexical activation; remove an ordering cue from the reconstruction prompt; replace the imagery framing with neutral wording; offer an explicit “no dominant direction” response option; and substitute a plain relational question for the gaze report, bringing the total to 14,900 responses.

The state of the question can be summarized as follows. It is established that human comprehension of spatial descriptions recruits the oculomotor system, and that this happens without any directional word being uttered (Altmann, 2004; Richardson & Spivey, 2000; Spivey & Geng, 2001). It is also established that language models reproduce a range of human-like behavioral signatures on linguistic tasks, and that such behavioral agreement is weak evidence about internal mechanisms (Linzen, 2020; Marcus & Davis, 2020; Warstadt et al., 2020). What remains unclear is narrower and prior to any mechanistic claim: when a text-only model answers a spatial question correctly, is it responding to the configuration the passage describes or to the directional vocabulary the passage contains? Existing evaluations of visual and spatial cognition in language and vision–language models establish that performance is uneven across tasks, but they rarely separate the two, because their items supply layout and directional wording together (Schulze Buschoff et al., 2025). The contribution of this study is a design that pulls them apart. Holding the described layout fixed across four linguistic versions, and reading out the same layout through four response formats that fail in different ways, yields a pattern that neither a pure lexical-echo account nor a strong spatial-representation account predicts. It also yields a methodological result we did not anticipate. Three of the ways in which the models initially appeared to fall short of recovering the layout turned out, under the control conditions, to be properties of how they were asked rather than of what they recovered, which is a caution that generalizes beyond this paradigm.

## 2. Related Work

The investigation of the cognitive abilities of language models is rooted in interdisciplinary research. In cognitive psychology, the attention network theory proposed by Posner and Petersen decomposes human attention into three functional components—alerting, orienting, and executive control—thereby providing a core framework for computational modeling (Petersen & Posner, 2012; Posner & Petersen, 1990). Biological spatial attention is grounded in neural systems for sensory processing and spatial orienting, whereas current language models lack direct sensorimotor embodiment (O’Regan & Noë, 2001; Petersen & Posner, 2012). The Transformer architecture represents a major breakthrough in artificial intelligence, as its self-attention mechanism enables the integration of global information (Vaswani et al., 2017). The distribution of attention weights in Transformers appears superficially similar to human selective attention, because the model dynamically adjusts its “focus intensity” by computing correlations among word vectors. However, whether this mathematical operation is equivalent to attention in the cognitive sense remains fundamentally questionable, since language models have neither perceptual organs nor intention-driven attentional control (Lindsay, 2021).

In research on vision–language associations, Kosslyn’s theory of mental imagery suggests that humans deepen language comprehension through spatial simulation (Kosslyn, 1994). The most direct behavioral evidence for this coupling comes from Spivey and Geng, who had participants listen to descriptions of scenes unfolding along a single axis, such as a rappeller descending a canyon wall or a train stretching to the left, while facing a blank screen (Spivey & Geng, 2001). Even with nothing to see, participants produced significantly more eye movements in the direction implied by the described layout, and the same effect appears when the visual world is removed after a scene has been established (Altmann, 2004; Richardson & Spivey, 2000). Crucially, the original stimuli never name the direction of gaze; the directional bias emerges from the spatial configuration alone, which is what makes the paradigm a test of spatial modeling rather than of directional vocabulary. Computational studies have shown that pretrained language models can exhibit human-like content effects in reasoning tasks, suggesting that they encode certain regularities from linguistic experience (Lampinen et al., 2024). However, behavioral similarity between language models and humans does not by itself demonstrate equivalence in their internal representations or processing mechanisms.

In terms of evaluation methodology, the validation of the cognitive plausibility of language models has mainly relied on behavioral-matching paradigms. Linzen’s syntactic sensitivity test shows that models can capture hierarchical structures (Linzen, 2020), while Warstadt et al. found that models exhibit human-like attentional biases in grammatical and semantic judgments (Warstadt et al., 2020). Nevertheless, such approaches have a fundamental limitation: behavioral similarity cannot prove equivalence in internal processing mechanisms (Lampinen et al., 2024; Linzen, 2020; Marcus & Davis, 2020). Eye tracking, as a gold-standard method in cognitive research, also faces challenges when adapted to artificial intelligence (Rayner, 2009). Abnar and Zuidema attempted to transform attention weights into “pseudo eye-movement data”; their results suggest that shallow layers of Transformer models tend to show local focusing, whereas deeper layers tend toward global diffusion (Abnar & Zuidema, 2020). This pattern differs from the human eye-movement process, which typically develops from local to global processing (Furlan & Smith, 2016; Guo et al., 2022; Horn & Leigh, 2011). More importantly, the traditional indicators of the reading eye—fixations, the relatively stable pauses during which information is acquired and whose duration scales with processing difficulty (Inhoff & Rayner, 1986; Sereno & Rayner, 2003); saccades, the rapid movements between fixations whose direction and amplitude reflect the scanning strategy (Radach & Kennedy, 2004); and regressions, movements back to already-visited locations that index comprehension difficulty (Schotter et al., 2014)—lack clear computational equivalents in language models (Jain & Wallace, 2019).

A more recent body of work asks directly how much spatial competence current models have, and its findings are strikingly uneven. Evaluating frontier models on classic spatial-cognition tasks such as mental rotation, perspective taking, and cognitive mapping, Ramakrishnan et al. (2025) reported performance near chance and below animal baselines, concluding that spatial intelligence has not emerged at scale. Broad batteries of visual-cognition tasks administered to multimodal models find a similar unevenness, with competence on some capacities and little on others (Schulze Buschoff et al., 2025). Closest to the present study, Wicke and Wachowiak (2024) adapted a psycholinguistic paradigm to test whether language and vision models share human intuitions about spatial schemas and found partial and inconsistent alignment. Set beside those results, the near-ceiling layout recovery we report below should be read narrowly. It concerns a text-only task in which the layout is stated in the passage and needs only to be tracked, not a task requiring transformation of a spatial representation, and the contrast suggests that spatial competence in these models is strongly task-dependent rather than uniformly present or uniformly absent.

Two further strands bear on how such competence should be measured. Work on prompt-induced response bias shows that the wording of a question, the order of the options, and the composition of the answer set can move a model’s answers substantially, independently of the content being probed. Few-shot accuracy varies from near chance to near state of the art with the format and ordering of the prompt, an instability traced to a prior bias towards particular answers (Zhao et al., 2021), and in multiple-choice formats, models favor particular options in ways that survive changes in content so that simply permuting the alternatives shifts measured accuracy (Zheng et al., 2024). A four-way forced choice over directions is exactly such a format, which is why the present design varies the response format deliberately and reports what happens when an indeterminate option is added to the answer set. And the embodied and multimodal literature supplies the reason for caution about the construct itself: the human effect we borrow is oculomotor, arising in a system with an eye and a motor plan (O’Regan & Noë, 2001; Spivey & Geng, 2001), whereas a text-only model produces only a verbal report. Verbal gaze reports are therefore an informative but indirect operationalization. They can establish what spatial information a model’s outputs are sensitive to, which is the question we ask, and they cannot establish that anything gaze-like occurs, which is a question we do not.

These methodological difficulties motivate the approach adopted in the present study. Rather than trying to read spatial cognition out of internal attention weights, we treat the model as a black box and observe its overt verbal behavior on spatial-imagery tasks derived from the Spivey and Geng paradigm. The central methodological move is to hold the described spatial layout constant while manipulating the linguistic cues that accompany it so that reliance on directional vocabulary can be dissociated from spatial modeling. Removing directional words probes whether a model can recover a layout from configuration alone; scrambling sentence order probes whether it depends on the narrative sequence in which the layout is presented; and inserting a directional word that contradicts the layout sets the two cues in competition and asks which the response follows. Combining these manipulations with a forced-choice direction report, a free-form gaze narration, and an explicit two-dimensional reconstruction yields quantifiable and interpretable indicators of spatial-simulation behavior while avoiding claims about mechanisms that generative behavior cannot license.

## 3. Materials and Methods

### 3.1. Eye Movements as a Window on Attention

During reading and scene perception, eye movements have long served as a window on the covert allocation of attention, since where and how long the eyes dwell reflects what is being processed rather than only what is visible (Duchowski, 2017; Rayner, 2009). What matters for the present work is that this gaze is not driven by the visual input alone: when a reader imagines a described scene, the same oculomotor system produces movements that trace the imagined layout even with nothing to see (Altmann, 2004; Richardson & Spivey, 2000; Spivey & Geng, 2001). A text-only language model, however, has no oculomotor system and none of the fixation-, saccade-, or regression-level indices of human reading, so we do not attempt to record its “eye movements” directly. Instead we probe this imagery-driven, language-mediated gaze behaviorally, asking whether a model can report, narrate, and reconstruct the spatial layout that such gaze would trace.

### 3.2. Overview

**Figure 1:**
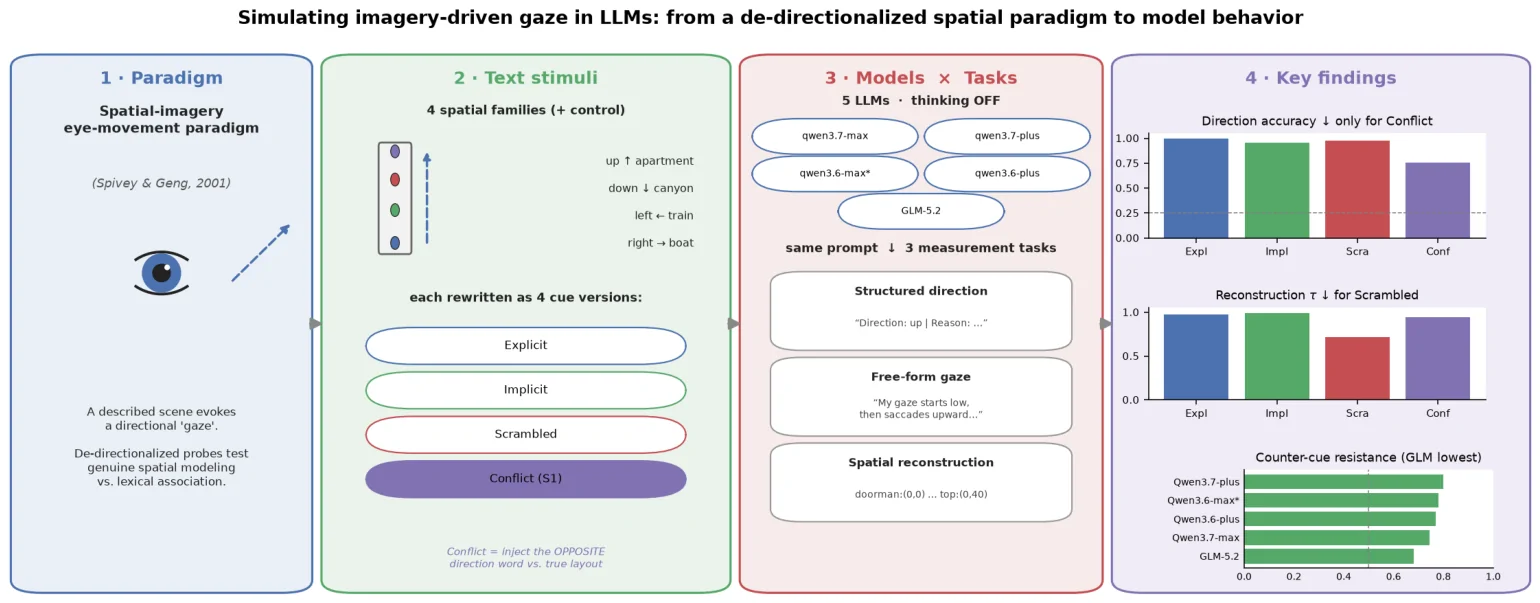
Overview of the study. A spatial-imagery paradigm adapted from Spivey and Geng defines four spatial families and non-directional controls; each directional scene is rewritten into four cue versions that hold layout constant while varying linguistic support; five language models are probed with three measurement tasks; and the resulting behavior is summarized along direction accuracy, spatial-order preservation, and counter-cue resistance (Spivey & Geng, 2001). The asterisk in model labels denotes the preview version, Qwen3.6-max-preview. Ellipses indicate intentionally abbreviated response examples. Commas within coordinate pairs separate the horizontal and vertical coordinates. Dashed arrows illustrate the direction of imagined gaze or scene traversal; the dashed horizontal line in the direction-accuracy panel marks four-alternative chance (0.25), and the dashed vertical line in the resistance panel marks 0.50 as a visual reference. The figure depicts the core design; additional control conditions extend it with four further insertion conditions, four further control scenes, and four additional response formats, as described in Section 3.5.2 and Section 3.6.

Our design adapts the spatial-imagery paradigm of Spivey and Geng (Spivey & Geng, 2001) to a purely linguistic setting. The logic, summarized in Figure 1, is to hold the described spatial layout of a scene constant while systematically weakening or contradicting the linguistic cues that would let a model answer without modeling that layout. Each scene is presented in several cue versions and probed with three measurement tasks so that a model that merely echoes directional words can be separated from one whose responses track the described configuration. All stimuli, prompts, and analyses are in English.

### 3.3. Hypotheses, Predictions, and Analysis Units

The design yields one primary hypothesis and three secondary ones, each with a directional prediction and a decision rule fixed before the analyses reported below. Table 1 states them together with the contrast that adjudicates each and the status of that contrast as confirmatory or exploratory. The primary hypothesis concerns whether responses are governed by the described configuration or by directional vocabulary. It is adjudicated by the absolute level of performance on the implicit passages, which never state the direction of gaze, together with the implicit-versus-scrambled contrast, which is a strict minimal pair of the kind documented in Section 3.5.1. It is deliberately not adjudicated by the implicit-versus-explicit contrast, because the audit reported in that section shows the explicit version is not matched to the implicit one on length or sentence structure; the explicit condition serves only as a descriptive ceiling. The secondary hypotheses concern the strength of that governance under a competing lexical cue, the dissociation between a categorical direction report and a relational reconstruction, and the presence of format-induced response biases.

Two features of the design constrain what can be tested. First, the independent experimental units are the model-by-scene cells, of which there are five times eight, or forty, for the directional stimuli. The repeated generations within a cell are stochastic repetitions of the same condition on the same item by the same system; they enlarge the precision with which a cell mean is estimated but they do not add independent units, and every inferential statement below is therefore computed over cells rather than over responses. Second, with eight scenes and five models the study is powered to detect large, consistent shifts and not small ones. We accordingly report it as an exploratory proof of concept: interval estimates are given for every quantity, the intervals are wide, and no claim rests on a contrast that is significant only when responses are treated as independent. One clarification about the confirmatory label in Table 1 is owed to the reader. The study was not preregistered, and the hypotheses and decision rules were fixed before the analyses reported here but after data collection. “Confirmatory” therefore distinguishes contrasts that were specified in advance of testing from those we report because they are informative, and it should not be read as the stronger guarantee that a preregistered design would provide.

### 3.4. Models

We evaluated five contemporary large language models accessed through Alibaba Cloud’s DashScope (Bailian) platform via its OpenAI-compatible API endpoint: Qwen3.7-max, Qwen3.7-plus, Qwen3.6-max-preview, and Qwen3.6-plus from the Qwen family, together with GLM-5.2 from the Zhipu family, the last served through the same endpoint. Using a single hosted API means that each model was queried in its deployed configuration rather than from local weights, which fixes the served model version but leaves provider-side implementation details outside our control. These are text-only conversational models, which is appropriate to our question because the Spivey and Geng effect is itself driven by language rather than by concurrent visual input. To obtain directly comparable outputs across models, the optional reasoning (“thinking”) mode of each model was disabled. Disabling that mode suppresses the emission of an explicit reasoning trace; it does not license any claim about the amount of internal computation that precedes a response, which remains unobserved. Sampling used a temperature of 0.7 and a nucleus-sampling threshold of 0.9. These settings leave the models close to deterministic on this task, which matters for how the repeated generations should be read: across the 160 forced-choice cells of twenty generations each, 88.1% returned an identical answer every time, and the modal answer accounted for 97.3% of generations in the average cell. The dispersion that does exist is concentrated where the models are genuinely divided rather than spread evenly. Every explicit-condition cell was unanimous, against 87.5% of implicit cells and 75.0% of counter-cue cells; in the reconstruction task the within-cell standard deviation of τ averaged 0.018 under explicit passages and 0.097 under scrambled ones. Sampling noise is therefore small relative to the differences between conditions reported below, and a lower temperature would be expected to sharpen those differences rather than to alter their ordering. No system message was supplied, no token limit was imposed, and no stop sequences were used, so each response is the model’s unconstrained completion of a single user turn. No random seed was supplied with the requests, so runs are reproducible only in distribution and not response by response; this is one reason the raw responses are deposited, in the public repository given in the Data Availability Statement, rather than only the derived summaries. Each request was issued independently, with no conversation history carried between requests, so repeated samples of the same stimulus are exchangeable within a cell. Failed requests were retried up to six times with exponential backoff, extended to a jittered backoff of up to sixty seconds on rate-limit responses, and a hard wall-clock cap of 165 s per request prevented stalled streams from blocking collection. Responses were collected between July and August 2026. After retries the failure rate was 0 of 8200 requests in the control round; in the main round every cell reached its target sample count, and only successfully returned responses enter the analyses. Spanning two model families and two generations allows us to ask whether the qualitative pattern of spatial behavior is a property of a single system or a more general characteristic of current models.

### 3.5. Stimulus Design

The stimulus set is organized around two orthogonal factors, the spatial direction of a scene and the linguistic cue version in which it is expressed. Four directions were used, corresponding to scenes that unfold predominantly upward, downward, leftward, and rightward. Each direction is instantiated by two scenes, one adapted from the original Spivey and Geng materials and one newly written for this study; the novel counterpart guards against the possibility that a model reproduces the expected direction by having memorized the source texts rather than by modeling the described space. Two non-directional scenes serve as controls, describing objects that have no dominant spatial axis. The families and their salient objects, listed in the order in which the scene traverses them, are given in Table 2.

Every directional scene was rewritten into four cue versions that leave the described spatial layout unchanged but vary the linguistic support for it. The explicit version states the direction of gaze in words, providing a surface cue that can be echoed without any spatial modeling. The implicit version is de-directionalized: it conveys only the spatial configuration and never names the direction of gaze, reproducing the original Spivey and Geng stimuli verbatim for the adapted families so that model behavior can be compared directly against the human data. The scrambled version contains exactly the same sentences as the implicit version but reordered so that the narrative no longer traverses the layout monotonically, which tests whether a model depends on the sequence in which the layout is presented rather than on the layout itself. The counter-cue version prepends a directional sentence that points against the true layout, placing a misleading lexical cue in direct competition with the spatial configuration. The upward apartment family illustrates the manipulation. Its implicit version reads

Imagine that you are standing across the street from a 40 story apartment building. At the bottom there is a doorman in blue. On the 10th floor, a woman is hanging her laundry out the window. On the 29th floor, two kids are sitting on the fire escape smoking cigarettes. On the very top floor, two people are screaming.

The explicit version adds that the gaze travels upward, the scrambled version presents the same floor descriptions out of order, and the counter-cue version opens with the misleading sentence “As you take it in, your gaze drifts downward” before the unchanged layout. In every version the ground-truth direction remains the true spatial direction of the scene, so a model that follows the inserted word in the counter-cue version answers incorrectly. Crossing eight directional scenes with four cue versions and adding the two single-version controls yields thirty-four stimulus items. The complete set of scene texts is provided in Appendix A.

#### 3.5.1. How Closely the Cue Versions Are Matched

Because a difference between two cue versions can only be attributed to the intended manipulation if nothing else differs, we audited the four versions sentence by sentence across all eight directional scenes. Two of the three comparisons are exact minimal pairs and one is not.

The scrambled version is, in every scene, a strict permutation of the implicit version: the multiset of sentences is identical, each sentence is reproduced verbatim, and only the order differs. Lexical content, sentence-level syntax, sentence length distribution, and passage length are therefore held constant by construction, and the contrast isolates presentation order. The counter-cue version is, in every scene, the implicit version with exactly one sentence prepended and nothing else altered, so that contrast isolates the inserted sentence. What the scrambling manipulation cannot hold constant is discourse coherence and the resolution of anaphora, since reordering sentences necessarily disturbs both. That is a property of the manipulation rather than an oversight in its implementation: no reordering can leave coherence intact while changing order. We therefore treat the scrambled condition as supporting rather than decisive evidence and return to this limitation in Section 5.

The explicit version is not a minimal pair. It was written as a compressed paraphrase that names the direction, and it averages 39.8 words against 53.0 for the implicit version, is shorter in every scene by between four and twenty-six words, and is in no scene a superset of the implicit wording. Sentence structure, information density, and discourse organization therefore all differ alongside the presence of a directional word. We accordingly draw no inference from the explicit-versus-implicit contrast, and it plays no role in any conclusion; it is retained only as a descriptive ceiling reference showing that, when the direction is stated outright, every model reports it.

One further property of the implicit passages needs to be stated precisely, because the natural shorthand for them overstates the case. What the implicit version withholds is a statement of where the gaze travels; it is not free of directional vocabulary, and it could not be, since a layout along an axis can hardly be described without axis terms. Scanning the eight implicit passages for the words *up*, *down*, *left*, and *right* together with their *-ward* forms, three contain one that agrees with the layout: the canyon passage has the climbers rappel down the wall, the train passage has the train extend to the left, and the boat passage has the rod extend to the right. Four contain one that points the other way: the shelf passage places the reader at the right end of a leftward shelf, the corridor passage at the left end of a rightward corridor, the boat is described as facing leftward, and the train’s engine as pointed to the right. Only the two upward passages and the well passage contain none at all.

This is worth reporting rather than glossing because the accuracies fall out in the direction opposite to a lexical-echo account. The five implicit passages that contain no direction word agreeing with the layout were answered correctly on every one of their 500 forced-choice trials, including the 2 whose only directional vocabulary contradicts the layout. The three that do contain an agreeing word average 0.873, and the one passage in the whole implicit condition that falls below ceiling is the train at 0.620, whose 38 errors are all responses of “right”—the direction of the engine’s orientation, which the passage names, and not of the layout. Where directional vocabulary and layout can be separated within the implicit condition, then, the responses follow the layout, and the residual errors follow a word only when that word is the one that disagrees with it.

#### 3.5.2. Additional Control Conditions

Four further insertion conditions were constructed to decompose the counter-cue manipulation, each again the implicit version plus exactly one sentence. The congruent condition inserts a directional sentence that agrees with the true layout, which holds the act of insertion constant while removing the conflict. The final-position condition places the original misleading sentence after the layout rather than before it. The reworded condition states the misleading direction in a declarative register that is not phrased as an instruction, for example, “Note that the overall movement here runs downward,” which separates directional content from instruction-like phrasing. The non-spatial condition uses the same misleading direction word in a context that makes no spatial assertion about the scene, for example, “As you take it in, you recall that local politics had been drifting rightward for years,” which separates lexical activation of a direction word from an assertion about where the gaze travels. Four additional non-directional control scenes were also written, describing objects arranged in a ring, a pile, a packed interior, and an overlapping cluster, because the two original controls proved unsatisfactory: the telescope scene implies a zoom hierarchy and the desktop scene leaves the layout underspecified. These additions bring the stimulus set to seventy items: thirty-two directional passages in the four original cue versions, thirty-two in the four new insertion conditions, and six non-directional controls.

### 3.6. Measurement Tasks

Each stimulus was probed with three tasks that read out the described layout in different ways, and that can fail in different ways. The forced-choice direction task asks the model to imagine the scene and report the single dominant direction of its gaze in a fixed format, which yields the primary accuracy measure:

You are in an imagination experiment. Read the scene below, imagine it with your eyes closed, and report the main direction your gaze moves. Choose exactly one of [up, down, left, right] and give a one-sentence reason. Answer strictly in this format, nothing else: Direction: X; Reason: …

The free-form task removes the answer template and instead asks the model to “naturally describe how your gaze moves as you imagine it, and why,” which exposes the spontaneous scanning pattern and the order in which objects are visited, without the standardizing pressure of a fixed format. The reconstruction task asks the model to place the named objects of the scene on a two-dimensional grid, with the horizontal axis positive to the right and the vertical axis positive upward, reporting one integer coordinate pair per object. This last task probes the spatial arrangement the model attributes to the scene rather than a summary direction, because the correctness of a reconstruction depends on the relative ordering of all objects along the dominant axis. The forced-choice task was sampled twenty times per stimulus and the free-form and reconstruction tasks ten times each. The reconstruction task was not administered to the two control scenes, whose objects have no dominant spatial order, so the three tasks together produced 6700 responses. The exact prompt templates and representative model responses for each task are given in Appendix B.

The four insertion conditions of Section 3.5.2, administered in the forced-choice format, contribute 3200 further responses. Four additional response formats contribute 5000 more, bringing the total to 14,900. The first of the four repairs a confound in the reconstruction prompt. That prompt named the objects to be placed, and it listed them in their correct spatial order, so a model could in principle have produced a well-ordered reconstruction by mapping list position onto the axis without consulting the scene at all. We therefore reran the task with the object list independently shuffled for every sample, using a fixed per-sample seed so that the shuffles are reproducible, and we report the shuffled version as the measure of record. The second removes the imagery framing from the forced-choice prompt: the phrases “imagination experiment,” “with your eyes closed,” and “your gaze moves” are replaced with neutral wording asking which direction the described scene predominantly runs, while the four response alternatives and the answer format are left untouched, so any difference isolates the framing. The third adds a fifth alternative stating that the description has no dominant direction, which removes the obligation to name one. The fourth abandons the gaze framing altogether and asks a plain relational question about the scene, namely, where the last-introduced object sits relative to the first-introduced one, scored against the ground-truth relation. Because this last format asks about the described world rather than about the model’s own imagined viewing behavior, it provides a check on whether the effects of interest depend on the introspective framing that the primary task adopts.

### 3.7. Scoring and Analysis

For the forced-choice task, the reported direction was read from the fixed answer field with a regular expression; when that field was absent the first directional word in the response was taken instead, and a response with no directional word was recorded as unclear. Because the answer format is rigid, this extraction is close to error-free, and the forced-choice measure is the one on which the conclusions rest.

For the reconstruction task, we quantified how well the model preserved the spatial order of the scene by computing Kendall’s τ between the ordering of the objects along the dominant axis implied by the model’s coordinates and the ground-truth ordering so that a value near one indicates a correctly ordered layout and a value near zero or below indicates a disordered or reversed one. Because a coordinate list can be degenerate in ways a single coefficient hides, we audited all 1600 reconstructions. Every one yielded at least two placeable objects and so entered the analysis, but 200 omitted one or more of the named objects and were scored on those present, 176 assigned two or more objects the same coordinate on the dominant axis, which τ treats as a tie rather than as an ordering error, and 24 assigned two objects identical coordinate pairs. Ties were distributed across all five models, from 24 to 43 of 320 reconstructions each, so they reflect a general tendency to place objects on a coarse grid rather than a single model’s quirk. These figures qualify the near-ceiling τ values reported below: a reconstruction can be free of ordering violations while still being spatially coarse, and τ rewards the former without penalizing the latter.

For the counter-cue version, each forced-choice response was classified as resisting the misleading word when it matched the true spatial layout, following the word when it matched the inserted opposite direction, or other otherwise. We designed this as a measure of how firmly a spatial commitment withstands a competing lexical cue, and Section 4.3 shows that it is not one; it is reported here as the proportion of responses matching each of the two competing cues, which is what it in fact records. The control scenes have no correct direction, so for these we report the distribution of spontaneously chosen directions as a false-alarm baseline that reveals any default response bias.

#### 3.7.1. Coding the Free-Form Narrations

The free-form task does not yield a single direction as readily as the forced-choice format. One candidate readout assigned each narration a single dominant direction by counting directional expressions and taking the most frequent. A narration containing no directional expression, or two directions tied for the highest count, was recorded as unclear and counted as incorrect. This is not a rare outcome, and its frequency is itself informative: 17.6% of the 1600 narrations were unclear under this rule, ranging from 7.0% under explicit passages to 22.2% under scrambled ones. Part of the apparent decline in free-form accuracy across cue versions is therefore a decline in how often the rule resolves at all, rather than a decline in how often it resolves correctly. To test that readout we wrote a second, deliberately different parser, which removes non-spatial idioms such as “right away” and “narrows down,” requires a directional word to occur within a short window of gaze or motion language rather than counting bare occurrences, and resolves by the last qualifying mention rather than by frequency. The two parsers agree on only 60.5% of the 1600 narrations, or 73.6% of those both resolve, with Cohen’s κ of 0.49. Inspection of the disagreements shows that most are not parser errors. The narrations typically describe non-monotonic scanpaths, snapping to a salient object, doubling back, and only then settling so that no single direction label is correct for them and any argmax rule imposes one. Direction-word coding is thus the wrong readout for an unconstrained narration, and we report it only as a descriptive figure.

We therefore adopt a readout that does not presuppose a single direction: the order in which the narration first mentions each of the scene’s salient objects, scored by Kendall’s τ against the ground-truth spatial order. This is the same quantity the reconstruction task measures, which makes the two directly comparable, and it remains well defined for a wandering scanpath. Objects were located with a fixed alias table, because narrations paraphrase freely, and a narration was scored only when at least three of the four objects could be located; this yielded 1165 scorable narrations of 1600, a coverage of 72.8%, with 3.17 objects located on average. The remaining narrations describe the scene too abstractly to be scored this way, and their exclusion is a limitation of the measure rather than a property of the models.

#### 3.7.2. Statistical Approach

All estimates and tests treat the model-by-scene cell as the unit of analysis, for the reasons given above. Interval estimates are two-way cluster bootstraps that resample scenes and models independently with replacement, using 4000 resamples, so that the intervals reflect uncertainty about which scenes and which models were sampled rather than only sampling noise within a cell. For the model-specific accuracy estimates, intervals instead resample scenes within each fixed model, using 2000 resamples. Conditions are compared with Wilcoxon signed-rank tests over the paired cell means, which requires no distributional assumption and respects the pairing of conditions within a cell. Because each task contributes six pairwise comparisons, *p* values are corrected within task using Holm’s step-down procedure, and we report both the raw and the corrected value throughout. As a convergent check that does not depend on aggregating to cell means, we also fitted binomial mixed-effects models to the individual responses with crossed random intercepts for model and for scene, estimated by variational Bayes; the explicit condition had to be excluded from these fits because accuracy was exactly one in every cell, which separates the data perfectly. Chi-square and two-proportion tests that treated individual responses as independent would overstate precision, and they are not used. Analyses were implemented in Python 3.11.5 using NumPy 2.0.0, pandas 2.2.3, SciPy 1.14.1, and statsmodels 0.14.0; the deposited figures were generated with Matplotlib 3.7.2.

## 4. Results

### 4.1. Directional Accuracy Across Cue Versions

**Figure 2:**
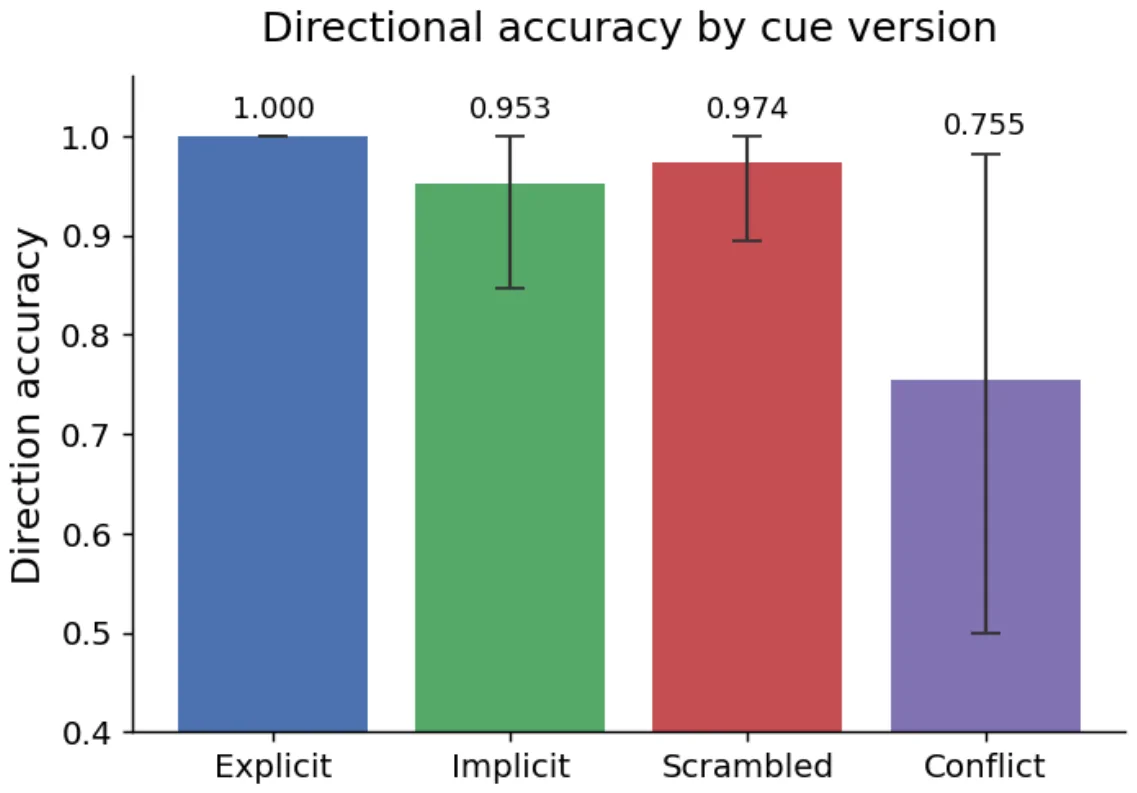
Forced-choice direction accuracy pooled across models for each cue version, with the exact value printed above each bar. Error bars are 95% two-way cluster-bootstrap intervals over scenes and models; the interval for the explicit condition is degenerate because accuracy is exactly one in every cell. The vertical axis is truncated at 0.4 to make the near-ceiling differences visible; four-alternative chance is 0.25, just below the plotted range. Accuracy is at ceiling with explicit cues, remains high after directional words are removed or sentences are reordered, and drops under counter-cue conflict.

The forced-choice direction task provides the primary test of whether models recover the layout of a described scene when the linguistic support for that layout is systematically varied. Pooling over the five models and the eight directional scenes, accuracy differed among cue versions, as summarized in Table 3 and Figure 2. With an explicit directional word present, every response matched the true layout (accuracy =1.000). Removing that word while leaving the spatial configuration intact reduced accuracy only modestly (implicit =0.953), and scrambling the sentence order left accuracy nearly as high (scrambled =0.974). Inserting a directional word that pointed against the true layout produced a qualitatively larger drop (conflict =0.755). Every cue version remained well above the four-alternative chance level of 0.25.

Once uncertainty is quantified over the forty model-by-scene cells rather than over individual responses, only one of these differences is reliable. The cluster-bootstrap intervals are wide: 1.000 with no spread for explicit, 0.953 with a 95% interval of [0.846,1.000] for implicit, 0.974 with [0.895,1.000] for scrambled, and 0.755 with [0.499,0.983] for conflict. Paired Wilcoxon tests across cells, Holm-corrected within the task, separate conflict from each of the other three versions (versus explicit, mean difference 0.245, $p_{\mathrm{Holm}}=0.002$; versus implicit, 0.198, $p_{\mathrm{Holm}}=0.002$; versus scrambled, 0.219, $p_{\mathrm{Holm}}=0.002$) but do not separate explicit, implicit, and scrambled from one another (explicit versus implicit, 0.048, $p_{\mathrm{Holm}}=0.129$; explicit versus scrambled, 0.026, $p_{\mathrm{Holm}}=0.136$; implicit versus scrambled, −0.021, $p_{\mathrm{Holm}}=0.362$).

If individual responses were treated as independent units, a chi-square test and two-proportion *z* tests would count twenty repeated generations of one item by one model as twenty independent observations and would separate every version from explicit at extreme significance levels. Those separations of explicit from implicit and from scrambled do not survive a correct treatment of the unit of analysis. The claim of interest is that removing the directional word, or reordering the sentences that convey the layout, does not degrade performance, and a non-significant difference is what that claim predicts; treating responses as independent would manufacture evidence against it. A binomial mixed-effects model fitted to the individual responses with crossed random intercepts for model and scene agrees on the large effect, placing conflict far below implicit (log-odds −2.93, 95% interval [−3.16,−2.69], odds ratio 0.054), but it places scrambled slightly above implicit with an interval excluding zero (log-odds 0.79, [0.33,1.25]). We give precedence to the cell-level test, both because the variational approximation used to fit the mixed model is known to understate posterior width and because a difference of two percentage points in the same direction as sampling noise does not warrant interpretation.

The pattern is informative in what it does and does not destroy. If models answered the direction task by echoing surface directional vocabulary, stripping those words in the implicit version should have collapsed performance; instead, accuracy remained above 0.95. If they depended on the narrative sequence in which the layout is presented, scrambling that sequence should have been catastrophic; instead, scrambled accuracy was indistinguishable from both explicit and implicit. The only manipulation that lowered accuracy was a lexical cue that contradicted the layout. In other words, the models’ forced-choice behavior is largely driven by the described spatial configuration, and a competing directional word is the one thing that displaces it. What that displacement actually indexes is taken up in Section 4.3, where the control conditions show it to depend almost entirely on where the competing word is placed rather than on how firmly the layout is held. Two qualifications belong here rather than in a later section. The explicit-versus-implicit comparison cannot carry the weight it appears to, because the two versions are not matched on length or sentence structure (Section 3.5.1); nothing in the argument depends on it, since the relevant claim is that implicit accuracy is high in absolute terms, not that it is statistically equal to a ceiling. And the scrambled manipulation necessarily disturbs discourse coherence along with sentence order, so its null result shows that the models are robust to the joint disruption, not that order specifically is irrelevant.

Separating the two stimulus sources reveals a difference that the pooled means conceal. On the four scenes taken from Spivey and Geng, accuracy was 1.000 for explicit, 0.905 for implicit, 0.982 for scrambled and 0.570 for conflict; on the four newly written scenes it was 1.000, 1.000, 0.965, and 0.940. The novel items are thus, if anything, easier, which argues against the possibility that performance on the implicit condition reflects memorization of the published Spivey and Geng texts: had the models been reciting remembered material, the borrowed items should have been the advantaged ones. The counter-cue effect, by contrast, is concentrated almost entirely on the borrowed items, a 0.37 difference in resistance that is larger than any effect of cue version on the novel items. The borrowed passages are longer, more clause-heavy, and more literary than the ones we wrote, so the most economical reading is that a misleading directional word competes more successfully when the layout is conveyed by denser prose. We return to this in Section 5, since it means the headline counter-cue effect rests on four scenes rather than eight.

**Figure 3:**
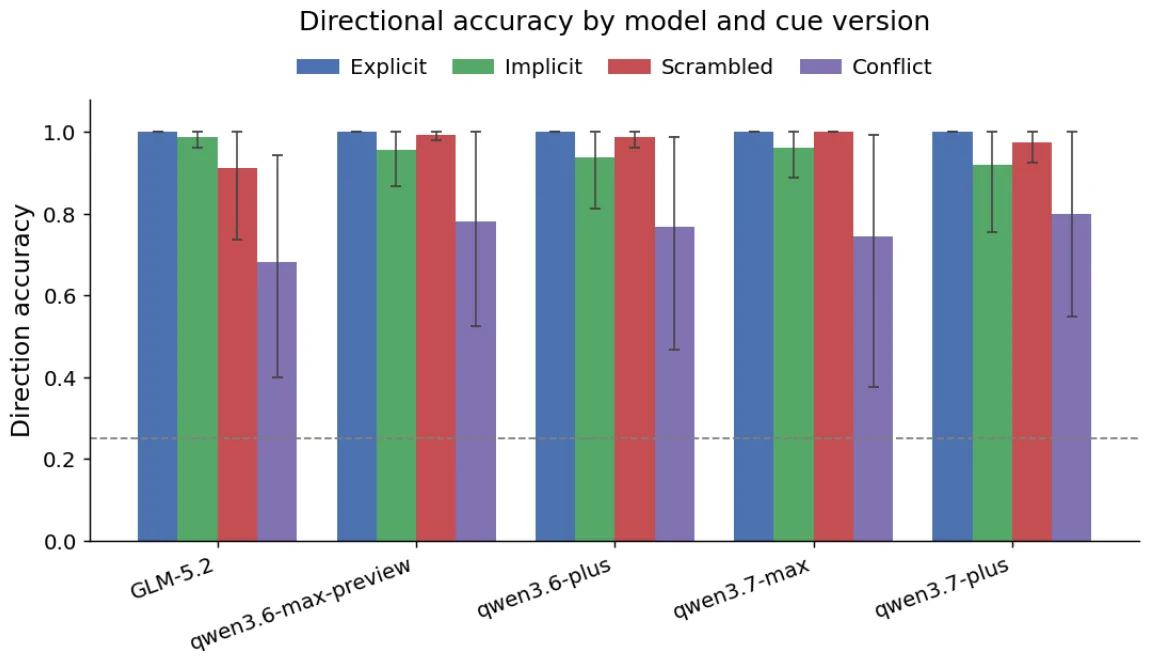
Forced-choice direction accuracy by model and cue version. Error bars are 95% bootstrap intervals obtained by resampling scenes within each model (2000 resamples). The dashed horizontal line marks four-alternative chance accuracy (0.25). Explicit performance is uniformly at ceiling; conflict is the only version that consistently lowers accuracy below 0.90 for every model.

The same dissociation appears at the level of individual models (Figure 3). Explicit accuracy was uniformly perfect. Implicit and scrambled accuracies were high for every model, with GLM-5.2 the most accurate under the implicit version (0.988) and the least accurate under the scrambled version (0.913), while Qwen3.7-max was perfect under scrambling. Conflict accuracy spanned a wider range, from 0.681 for GLM-5.2 to 0.800 for Qwen3.7-plus, indicating that models differ not so much in whether they can recover a layout as in how firmly they hold that layout against a misleading word. Within the Qwen family, the qualitative ordering of cue versions was shared across both generations, which suggests that the pattern is a property of current instruction-tuned language models rather than of a single release.

**Figure 4:**
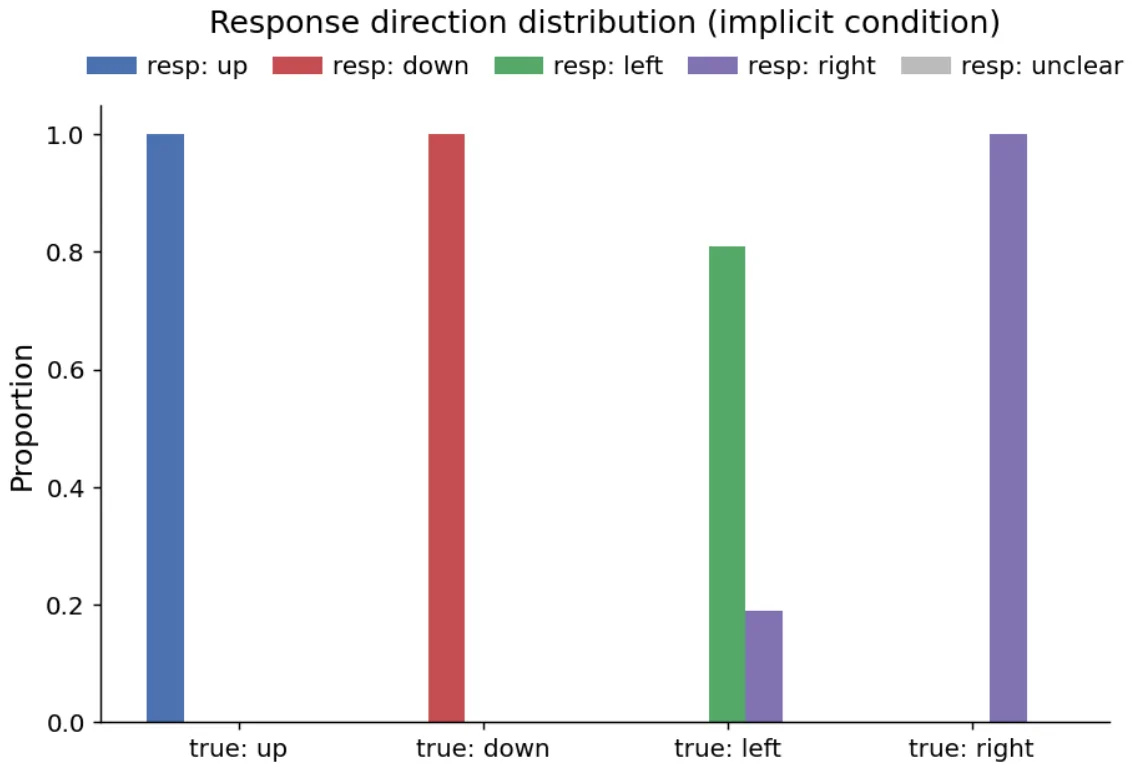
Distribution of forced-choice responses under the implicit condition, conditioned on the true direction of the scene. Upward, downward, and rightward scenes are recovered perfectly; leftward scenes are the only residual source of error, with a minority of responses reported as rightward. Those errors come from one of the two leftward scenes. The legend includes all response categories; the unclear category has zero observations, so no bar is visible for that category.

Inspection of the response distribution under the implicit condition further clarifies where residual errors arise (Figure 4). When the true direction was upward, downward, or rightward, every response matched that direction. The only systematic confusion was leftward scenes being answered as rightward on a minority of trials (approximately 19% of leftward responses). That confusion is confined to a single item. Seven of the eight directional scenes were answered correctly on all 100 of their implicit trials; the train alone fell to 0.620, and all 38 of its errors are responses of “right,” the direction in which its passage says the engine points. The residual error in the implicit condition is thus not a diffuse left–right weakness but the effect of one competing word in one passage. A weakness along the horizontal axis does appear on the non-gaze relational probe, where it is spread more widely: upward and downward relations were reported correctly on all 200 trials each, whereas leftward and rightward relations were correct on 0.870 of trials, with three of the four horizontal scenes below ceiling. This is consistent with responses being more reliable along axes that are richly lexicalized and frequently ordered in text (above versus below, top versus bottom) than along a left–right axis whose linguistic encoding is more contingent on viewpoint. Since that probe never mentions gaze, the asymmetry is a property of the axis rather than of the imagery framing.

### 4.2. Spatial Reconstruction

**Figure 5:**
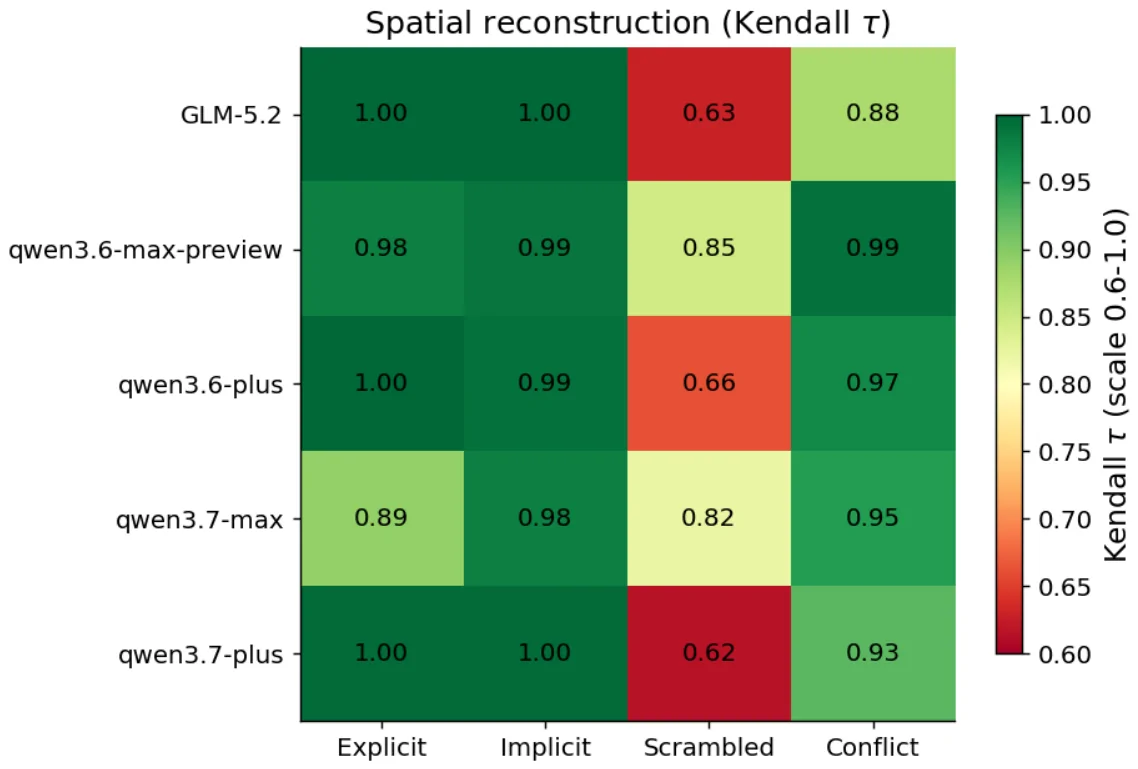
Reconstruction quality (Kendall’s τ) by model and cue version, with the exact value printed in each cell. The color scale spans τ=0.6 to 1.0 to emphasize the differences among conditions. Explicit, implicit, and conflict versions preserve spatial order; scrambled versions degrade it.

The reconstruction task asks whether the directional response observed above is accompanied by a correctly ordered placement of the scene’s objects or is merely a summary label. Mean Kendall’s τ between the object order implied by the model’s coordinates and the ground-truth order along the dominant axis is reported in Table 4 and visualized in Figure 5. Under the implicit version, reconstruction was essentially perfect (overall τ=0.991), and under the explicit version it remained very high (τ=0.973). Critically, the counter-cue version that had lowered forced-choice accuracy by nearly a quarter left reconstruction largely intact (τ=0.943). Scrambling sentence order, by contrast, produced the largest drop in reconstruction quality (τ=0.715).

Analyzed over cells, the intervals are [0.868,1.000] for explicit, [0.968,1.000] for implicit, [0.467,0.920] for scrambled, and [0.840,1.000] for conflict. Only the scrambled condition is reliably distinguished from the others (versus implicit, mean difference 0.276, $p_{\mathrm{Holm}}<0.001$; versus explicit, 0.258, $p_{\mathrm{Holm}}<0.001$; versus conflict, 0.228, $p_{\mathrm{Holm}}<0.001$), while explicit, implicit, and conflict are mutually indistinguishable (all $p_{\mathrm{Holm}}\geq 0.526$). The reconstruction result is therefore a single effect of sentence order, not a graded ordering of four conditions.

This result required a control before it could be believed. The prompt named the objects to be placed and listed them in their correct spatial order, so a model could have produced a well-ordered reconstruction by transcribing list position onto the axis without reading the scene at all, and the near-ceiling τ values would then measure nothing but compliance. Rerunning all 1600 reconstructions with the object list shuffled independently for each sample leaves the pattern intact: τ=0.984 for explicit, 0.960 for implicit, 0.746 for scrambled, and 0.898 for conflict, with no condition differing reliably from its ordered-list counterpart (largest change −0.046 under conflict, all $p_{\mathrm{Holm}}\geq 0.198$). The models were consequently not exploiting the list order, and we report the shuffled-list values as the measure of record. The reconstruction figures should nonetheless be read alongside the audit in Section 3.7: τ near one certifies the absence of ordering violations, and 176 of the 1600 reconstructions place two or more objects at the same coordinate on the dominant axis, which τ scores as a tie rather than as an error. Ordering is preserved; metric precision is not established.

This dissociation between the two tasks is theoretically important. A misleading directional word can pull the forced-choice report away from the true layout without destroying the relative ordering of the objects that the model places on the grid; conversely, destroying the narrative sequence in which those objects are introduced can scramble the reconstructed order while leaving the summary direction largely intact. The two measures therefore do not merely restate one another. Forced choice indexes a categorical commitment that is sensitive to competing lexical cues, whereas reconstruction indexes a relational structure that is more sensitive to the sequential organization of the description. A model can, in other words, still place the objects in their correct relative order even when a counter-cue has displaced its directional report, and it can still name the correct overall direction even when the order in which it places those objects has been disrupted by scrambling. Neither pattern would be expected if the only thing the models recovered from the scenes were isolated directional keywords.

Model differences again concentrated on the scrambled and conflict versions. Qwen3.6-max-preview retained the highest scrambled τ (0.846), whereas Qwen3.7-plus and GLM-5.2 fell to approximately 0.62. Under conflict, every Qwen model maintained τ above 0.92, while GLM-5.2 dropped to 0.875, echoing its greater susceptibility on the forced-choice conflict measure.

### 4.3. Counter-Cue Conflict: Word Versus Layout

**Figure 6:**
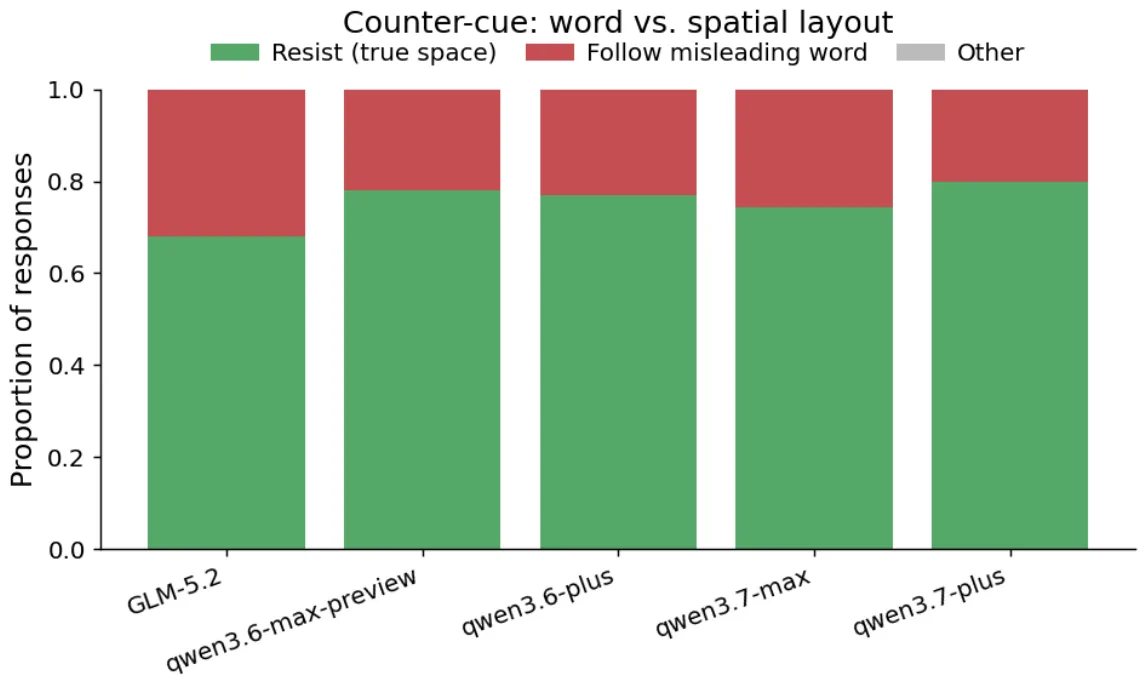
Proportion of counter-cue responses that resist the misleading word (true layout) versus follow it, by model, for the original sentence-initial placement of the cue. Resistance predominates in every model, but a non-negligible minority of responses follow the word, and GLM-5.2 is the least resistant. Figure 7 shows that this pattern reverses completely when the identical sentence is placed after the layout, so the differences among models here are small relative to the effect of cue position. The Other category denotes responses matching neither the true layout nor the misleading word; it has zero observations for every model, so no corresponding stacked segment is visible.

The conflict condition places a directional word in direct competition with the spatial configuration. Across 800 conflict trials, models resisted the misleading word and followed the true layout on 75.5% of responses, and they followed the word on the remaining 24.5% (Table 5; Figure 6). No response fell into a residual category that matched neither the layout nor the word, which indicates that the conflict is resolved as a binary choice between the two cues rather than as an unstructured failure. We present these figures first because they were the basis of our original claim, and we then show that they do not mean what we took them to mean: the control conditions below establish that this rate is determined by the position of the inserted sentence and by the direction it names, so it should not be read as an estimate of how firmly the models hold a spatial commitment.

Read on its own, the resistance rate of roughly three quarters appears to support a spatial-modeling interpretation: if the models answered solely by lexical association, the inserted directional word should have dominated. The interval around that estimate is already wide, however, at [0.499,0.983] over cells, and the additional control conditions show that the quantity is not a stable property of the models at all. It is a property of where the misleading sentence is placed and of which direction it names.

#### The Counter-Cue Effect Decomposed

**Figure 7:**
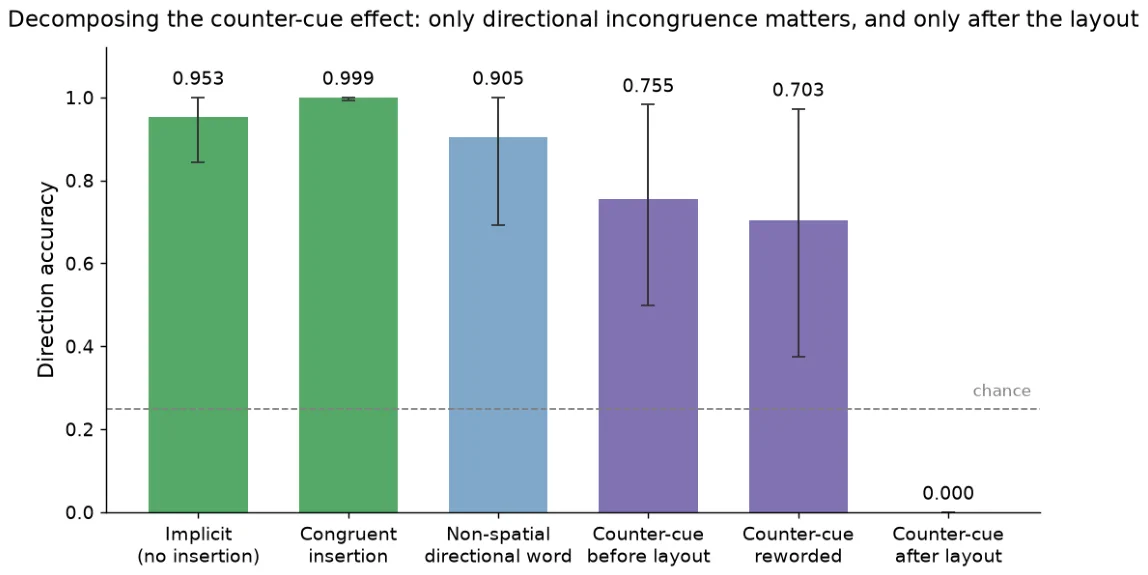
The counter-cue effect decomposed. Each condition is the implicit passage plus exactly one inserted sentence, so every bar is a minimal pair with the implicit baseline. A congruent insertion is harmless and a non-spatial use of the direction word nearly so; rewording the cue as a declarative changes little; moving the identical cue to the end of the passage eliminates correct responding entirely. Error bars are 95% two-way cluster-bootstrap intervals over scenes and models. The dashed horizontal line denotes four-alternative chance accuracy (0.25).

Four further insertion conditions, each the implicit passage plus exactly one sentence, separate the components that the original manipulation confounded (Figure 7). Inserting a sentence that agrees with the layout leaves accuracy at ceiling (0.999, interval [0.993,1.000]), indistinguishable from the implicit baseline of 0.953 ($p_{\mathrm{Holm}}=0.129$), so the act of inserting a sentence is not itself disruptive and the counter-cue effect is attributable to the contradiction rather than to the insertion. Using the misleading direction word in a plainly non-spatial sense—recalling that local politics had been drifting rightward—costs little (0.905, [0.696,1.000]; also indistinguishable from baseline, $p_{\mathrm{Holm}}=0.129$), though it is reliably less damaging than a genuine counter-cue ($p_{\mathrm{Holm}}=0.015$). Mere lexical activation of a direction word is therefore not what drives the effect; the sentence must assert something about the scene. Rewording the counter-cue from an instruction into a declarative statement changes nothing (0.703 against 0.755, $p_{\mathrm{Holm}}=0.146$), so the effect does not depend on the cue being phrased as an instruction to be obeyed.

The fourth condition overturns the headline number. Moving the identical misleading sentence from the beginning of the passage to the end drops accuracy from 0.755 to exactly zero: all 800 responses, in every scene and every model, followed the misleading word ($p_{\mathrm{Holm}}<10^{-5}$). Nothing about the sentence changed but its position. The apparent three-quarters resistance is thus an artifact of sentence-initial placement, where the cue arrives before the layout has been described and is presumably superseded by it; a cue arriving after the layout wins without exception. The data therefore do not support a general claim that the models resist misleading directional words. What the data support is narrower and, we think, more interesting: a directional assertion and a described layout compete, and recency decides the competition. A model reports the direction that the most recent relevant statement supports.

**Figure 8:**
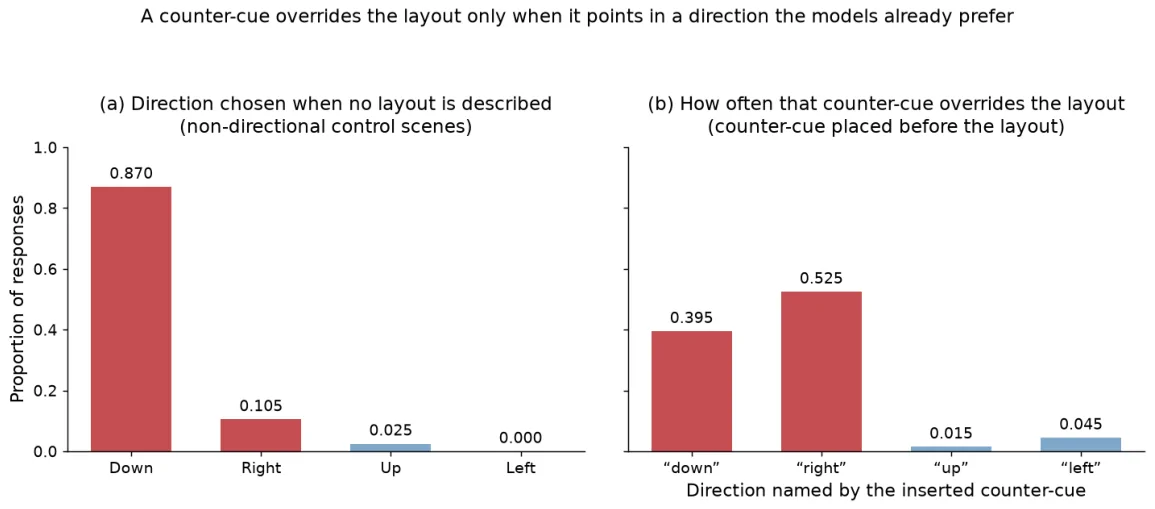
A misleading word succeeds mainly when it points where the models already tend to go. (**a**) The distribution of responses to non-directional control scenes, which supply no axis; (**b**) the rate at which a sentence-initial counter-cue is followed, by the direction the cue names. Downward and rightward, the two directions favored in the absence of any layout, are also the two in which a counter-cue is effective.

A second regularity constrains the interpretation further. Within the original sentence-initial condition, the counter-cue succeeded only when it pointed in particular directions. Its success rate was 0.525 when it named rightward and 0.395 when it named downward, against 0.045 for leftward and 0.015 for upward (200 trials each). Those are the same two directions the models default to when a scene supplies no axis at all, where 87.0% of responses were downward and 10.5% rightward with none leftward. Both sets correspond to the direction in which English text is read. A misleading word, in short, overrides the layout mainly when it points where the model was already disposed to go, which is a fact about response bias rather than about the strength of a spatial representation (Figure 8). Model differences are correspondingly minor next to these two effects: resistance ranged from 0.681 for GLM-5.2 to 0.800 for Qwen3.7-plus, a spread far smaller than the gap between initial and final placement of the same sentence. The effect is also unevenly distributed across items, falling almost entirely on the four passages borrowed from Spivey and Geng (0.570 resistance) rather than on the four we wrote (0.940).

### 4.4. Free-Form Narration

The free-form narration task removes the forced-choice template and asks the model to describe how its gaze moves through the imagined scene. Coding each narration with a single dominant direction gives 0.915 for explicit, 0.670 for implicit, 0.528 for scrambled, and 0.542 for conflict, each over 400 narrations with unresolved narrations counted as incorrect. We report these figures only as a descriptive contrast with the mention-order measure below; they should not be interpreted as a result. As Section 3.7 documents, two independently written parsers agree on only 60.5% of the narrations (κ=0.49), and the disagreements are mostly cases where the narration describes a genuinely non-monotonic scanpath to which no single direction label applies. The measure is unreliable, and the reliable part of it—the ordering of the four conditions—is preserved under the stricter parser but is also the part that carries least information.

Scoring the same narrations by the order in which they mention the scene’s objects gives a measure that does not presuppose a single direction, and the result is both cleaner and more informative. Mention-order τ was 0.853 for explicit ([0.668,1.000]), 0.871 for implicit ([0.744,0.984]) and 0.769 for conflict ([0.382,0.967]), none of which differ reliably from one another (all $p_{\mathrm{Holm}}=1.000$), against 0.397 for scrambled ([0.097,0.641]), which differs from all three (all $p_{\mathrm{Holm}}<0.001$). The narrations therefore traverse the objects in their correct spatial order whether the direction is stated, withheld, or actively contradicted, and they lose that order only when the sentences conveying the layout are reordered. This is the same pattern the reconstruction task produces, and on the same measure, which is reassuring: two response formats that fail in different ways agree that sentence order is what matters and that a contradicting word is not. Direction-word coding of the same narrations would take lower accuracy to mean that narration is less faithful to the layout. Narration is faithful to the layout; it is simply not reducible to one direction. As with the forced-choice data, the scrambling effect is concentrated on the borrowed passages (τ=0.105, against 0.540 for the novel ones). Coverage of this measure is 72.8% of narrations, the remainder describing the scene too abstractly to locate three objects in it.

### 4.5. Control Scenes

The two non-directional control scenes provide a complementary baseline. With no dominant spatial axis to recover, forced-choice responses were not uniformly distributed across the four alternatives: of 200 responses, 87.0% were downward, 10.5% rightward, and 2.5% upward, with no leftward responses. This strong downward default indicates that, in the absence of a layout, the models do not sample directions at random but fall back on a preferred response. Any interpretation of directional accuracy on the critical scenes must therefore be read against this baseline: the high accuracies observed for upward, leftward, and rightward layouts cannot be attributed to a general preference for those directions, because the control distribution itself is heavily biased toward down. The next section shows that this default is an artifact of the four-alternative response format rather than a judgement about the scenes, and Section 4.3 has already shown that the same two favored directions are the ones in which a counter-cue is effective. The control bias also underscores a broader point about construct validity. A model that produces a plausible gaze direction when a scene supplies one may still harbor strong response defaults when the scene does not, which is a further reason not to equate verbal gaze reports with the endogenous, stimulus-driven dynamics of human oculomotor control (Rayner, 2009; Spivey & Geng, 2001).

### 4.6. Robustness of the Response Format

**Figure 9:**
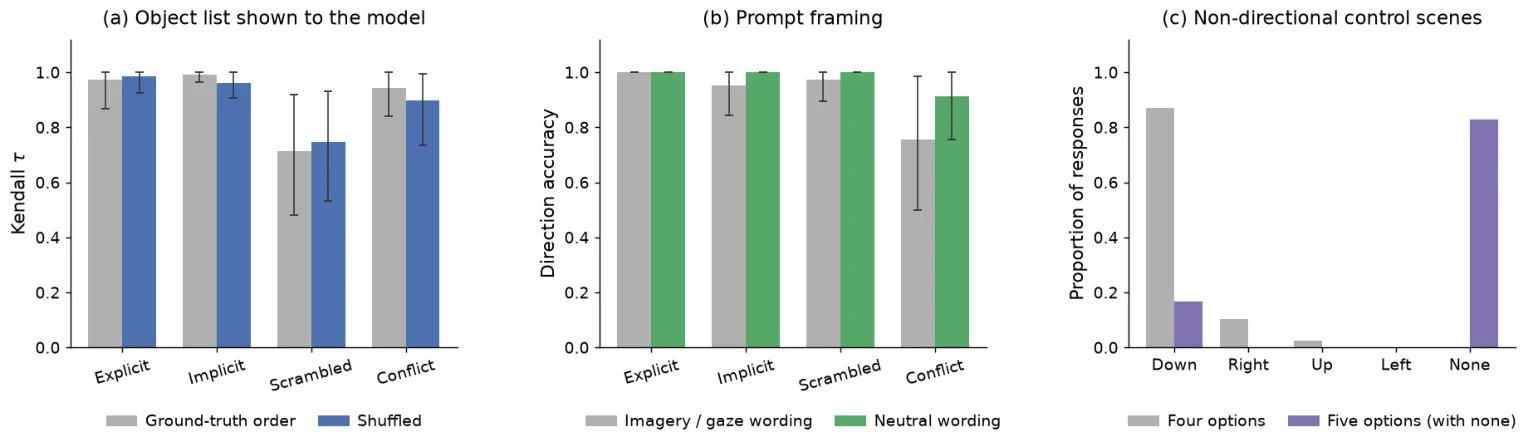
Three checks on the response format. (**a**) Reconstruction quality is unchanged when the object list in the prompt is shuffled rather than given in spatial order, ruling out the ordering leak. (**b**) Removing the imagery and gaze framing from the forced-choice prompt does not lower accuracy. (**c**) Offering an explicit “no dominant direction” option eliminates the downward default on control scenes while leaving accuracy on directional scenes unchanged; the four-option bars are the two original control scenes and the five-option bars pool all six, the matched two-scene comparison being given in the text. Error bars are 95% two-way cluster-bootstrap intervals over scenes and models.

Three further controls ask whether the primary findings depend on how the question was posed (Figure 9). Removing the imagery framing from the forced-choice prompt—dropping “imagination experiment,” “with your eyes closed,” and “your gaze moves” while leaving the alternatives and the answer format untouched—does not reduce accuracy and in fact raises it to exactly 1.000 for the explicit, implicit, and scrambled conditions. Under the counter-cue it rises from 0.755 to 0.913 ($p_{\mathrm{Holm}}=0.072$). The layout-recovery result is thus not an artifact of asking the model to introspect about its own gaze; if anything, the introspective framing invites the model to narrate a wandering attentional itinerary and thereby makes a misleading directional cue easier to adopt. Asking a plain relational question instead—where the last-introduced object sits relative to the first—yields 0.905 under implicit passages and 0.965 under counter-cue ones, confirming that the spatial relation survives a manipulation that disturbs the gaze report.

The remaining control concerns the response set. Offering a fifth alternative stating that the description has no dominant direction leaves accuracy on directional scenes unchanged at 0.950 (p=0.892 against the four-option version), and not one of the 400 responses took the new option, so the forced-choice measure was not inflated by models settling for a direction they would otherwise have declined to name. On the control scenes the option changes the picture entirely. Restricting the comparison to the two control scenes that were administered under both response sets so that the same items are compared under the two response sets, 96.0% of the 200 responses now state that there is no dominant direction and only 3.5% say downward, against the 87.0% downward observed when the same two scenes were presented with four options. Across all six control scenes, four of which are new, 83.0% of the 600 responses take the indeterminate option. The downward default reported above is therefore a consequence of the response format rather than a belief about the scenes, and it disappears once the models are permitted to decline. One control scene is an instructive exception. In a scene describing objects piled in a backpack, 65% of responses still chose downward even with the indeterminate option available, which is a defensible reading of a pile and suggests that this item was not as axis-free as intended. The other five scenes elicited between 82% and 100% indeterminate responses.

These format effects do not disturb the central result, which they leave intact or strengthen, but they bear directly on the interpretation of the counter-cue and control findings. Two phenomena that a single response format would attribute to the models’ spatial representations—a residual downward default and a partial vulnerability to misleading words—are substantially properties of the response format and the position of the cue.

## 5. Discussion

The central question of this study was whether the responses of contemporary language models to spatial descriptions are governed by the configuration those descriptions convey or by the directional vocabulary they contain, a question posed against the background of the imagery-driven gaze that Spivey and Geng documented in human comprehenders (Altmann, 2004; Richardson & Spivey, 2000; Spivey & Geng, 2001). The answer has two parts, and the second was not one we set out to look for. One finding is robust across every manipulation we tried. When the spatial layout is recoverable from configuration alone, with no statement anywhere in the passage of the direction the gaze travels, the models report the expected direction at near-ceiling accuracy, reconstruct the relative order of the scene’s objects with high fidelity, narrate the objects in their correct spatial order, and answer a plain relational question about them correctly. This holds when the directional word is absent, when the sentences are reordered, when the object list in the prompt is shuffled, when the imagery framing is stripped from the question, and when an escape option is offered. Responses to these passages are governed by the described configuration and not by directional vocabulary, and that is the study’s positive result.

A second finding is that most of the ways in which the models depart from this picture turn out, on closer inspection, to be properties of how they were asked rather than of what they represent. The apparent partial vulnerability to a misleading directional word is almost entirely a matter of where that word sits: three quarters of trials resist it before the layout is described, and none resist it afterwards. The apparent downward bias on scenes without an axis is a consequence of forcing a choice among four directions, and it vanishes when declining is permitted. The apparent unfaithfulness of free-form narration was an artifact of our own coding, which imposed one direction on descriptions of wandering scanpaths. Each correction removes a phenomenon we had attributed to the models and relocates it in the instrument. What remains, once they are removed, is a narrower claim: these models recover the spatial configuration of a described scene reliably, and the behavioral signatures that appeared to qualify that recovery were largely signatures of our measurements.

The dissociation between the categorical and the relational measures sharpens this interpretation. Inserting a counter-cue word lowered categorical direction accuracy substantially while leaving Kendall’s τ for reconstructed object order largely intact; scrambling sentence order did the reverse, degrading reconstruction while leaving direction accuracy near ceiling. The mention order of the narrations behaves like the reconstruction rather than like the direction report, falling under scrambling and holding under the counter-cue, so the split is not an idiosyncrasy of the coordinate task but a division between measures that ask for one label and measures that ask for an arrangement. A single underlying quantity cannot produce both patterns. Forced choice appears to index a summary commitment that is sensitive to competing lexical cues, whereas the order-based measures index a relational structure that is more sensitive to the sequential organization of the description. We note that this makes the categorical measure the more fragile of the two, which is the opposite of how such measures are usually treated: a single direction word is what evaluations of spatial reasoning most often ask a model to produce, and it is the readout most easily displaced by a competing word. This dissociation is consistent with the broader claim that language comprehension can engage an internal spatial model (Franklin & Tversky, 1990; Kosslyn, 1994), while also cautioning that different behavioral probes of that model need not move in lockstep. It further suggests that evaluations of “spatial reasoning” in language models that rely on a single report format risk mistaking format-sensitive categorical answers for deeper relational structure, or vice versa.

The conflict condition was designed as the critical test of the lexical-association confound, on the reasoning that a model tracking directional vocabulary should follow an inserted word pointing against the layout, whereas a model consulting a spatial representation should resist it. The control conditions show that this reasoning was too simple and that the question the conflict condition answers is not the one it was built to answer. Resistance is not a stable quantity that can be compared against a threshold. The same misleading sentence is resisted on three quarters of trials when it precedes the layout and on none at all when it follows it, so the design does not measure the strength of a spatial commitment; it measures which of two competing statements arrived more recently. That recency should decide the outcome is unsurprising for an autoregressive model, and it is not obviously a spatial fact about the model at all.

Two further observations point the same way. A counter-cue is effective almost exclusively when it names downward or rightward, the two directions the models fall back on when a scene affords no axis, and it is nearly inert when it names upward or leftward. And the effect is concentrated on the four longer, more literary passages borrowed from Spivey and Geng rather than on the four we wrote. What looked like a graded vulnerability of spatial representations to lexical interference is better described as the joint action of recency, a reading-order response bias, and passage difficulty. This is a less tidy conclusion than a graded-vulnerability account, but it is more useful, because each of these three factors is separately measurable and none of them requires positing a spatial representation of variable strength.

Model differences are correspondingly demoted. Resistance ranged from 0.681 for GLM-5.2 to 0.800 for Qwen3.7-plus, a spread of twelve points that is dwarfed by the seventy-five-point swing produced by moving one sentence within the prompt. Ranking systems on a measure that is this sensitive to a formatting choice would not be informative, and we do not do so.

The free-form and control results further qualify the claim that models “simulate gaze,” though not by showing them to be unfaithful to the layout. Narrated scanning patterns are frequently non-monotonic, attention being captured by a salient element before a more orderly traversal begins; such salience-driven itineraries are not in themselves uninteresting, since human gaze is likewise attracted by contrast and biological relevance (Petersen & Posner, 2012; Posner & Petersen, 1990). Scored by a single dominant direction, these narrations look unfaithful to the layout. Scored by the order in which they visit the scene’s objects, that reading is wrong: mention order tracks the true spatial order about as well under a contradicting word as under an explicit one, and it degrades only under scrambling. The narrations are faithful to the layout; what they are not is reducible to a single direction, and the apparent deficit was a property of our coding scheme. The control scenes make a related point about format. When no layout is available and four directions are the only options, responses collapse onto a downward default; when an explicit “no dominant direction” option is offered, five of six control scenes elicit that option on more than four fifths of trials, and accuracy on the directional scenes is unchanged. The default was therefore an artifact of a forced choice among four inapplicable alternatives rather than a spatial belief—a reminder that a constrained response format can manufacture the appearance of a systematic bias. Taken together with the observation that Transformer attention is a distribution over tokens rather than a spatially mapped sensorimotor act (Abnar & Zuidema, 2020; Lindsay, 2021; Vaswani et al., 2017), these findings recommend a cautious reading of the metaphor that opened this paper. Language models can open a window onto the spatial structure encoded in text, but that window is not the same as the human mind’s oculomotor window onto an imagined world.

### Limitations

Several limitations constrain how far these conclusions should be pressed. The most important is statistical power. Eight directional scenes and five models yield forty independent units, which is enough to detect the large effects reported here and not enough to detect small ones; the cluster-bootstrap intervals are correspondingly wide, spanning nearly half the scale in the counter-cue condition. The study should be read as an exploratory demonstration that a particular design separates layout from lexicon, not as a precise estimate of any quantity. A confirmatory version would need many more scenes, and scenes sampled rather than hand-written. Related to this, the counter-cue and scrambling effects are concentrated on the four passages borrowed from Spivey and Geng rather than on the four we wrote, so those effects rest on four items and may reflect properties of that prose—greater length, denser clause structure—rather than of the manipulation.

Three features of the materials limit specific inferences. The explicit condition is not matched to the implicit condition on length or sentence structure, so it functions only as a descriptive ceiling and supports no contrast. The scrambling manipulation necessarily disrupts discourse coherence and anaphora along with sentence order, so its effect cannot be attributed to order alone. And the reconstruction measure certifies ordering rather than metric accuracy: about one reconstruction in nine places two or more objects at the same coordinate on the dominant axis, which Kendall’s τ treats as a tie, so near-ceiling τ is compatible with spatially coarse placement.

The free-form narration remains the weakest measure. Direction-word coding of these narrations is unreliable, with κ=0.49 between two independent parsers, and although the mention-order measure that replaces it is well defined and yields a clear pattern, it can be computed for only 72.8% of narrations and depends on a hand-built alias table for locating objects in paraphrase.

The study examined five API-accessible conversational models from two families, with optional reasoning modes disabled; the results therefore speak to a particular slice of the current model landscape rather than to open-weight systems of every scale or to models that elaborate an extended chain of thought before answering. It also speaks to a particular slice of the task landscape, and this bears emphasis. Our scenes state a layout that need only be tracked; they do not require it to be transformed. On tasks that do—mental rotation, perspective taking, cognitive mapping—frontier models perform near chance and below animal baselines (Ramakrishnan et al., 2025), and adaptations of other psycholinguistic paradigms find only partial alignment with human spatial intuitions (Wicke & Wachowiak, 2024). The near-ceiling recovery reported here should not be generalized to spatial cognition broadly; read together with those results, it locates a narrow band of competence rather than establishing a general one. Because these are hosted services whose parameters we cannot inspect and whose versions may be retired, the responses are archived rather than assumed to be regenerable, and because no random seed was supplied with the requests, runs reproduce in distribution rather than response by response. The design holds the described layout constant while manipulating linguistic cues, but it does not include a concurrent human eye-tracking replication under the same rewritten stimuli; comparison with Spivey and Geng is therefore conceptual rather than trial-matched. Finally, all probes are verbal. Behavioral alignment on spatial-imagery tasks cannot, by itself, establish equivalence of internal mechanisms (Lampinen et al., 2024; Linzen, 2020), and nothing in the present data licenses claims about how spatial information is represented in the models’ activations. We use “recovers the configuration” throughout as a description of response behavior, with no implication that anything in the model resembles a spatial image.

These limitations also point toward productive next steps. A matched human eye-tracking study with the same cue versions would tighten the comparison between model behavior and the original imagery-driven gaze effect. The position effect deserves study in its own right, since a manipulation whose outcome inverts completely when one sentence is moved is a sharp tool for probing how autoregressive models arbitrate between competing assertions, and its interaction with extended reasoning is an obvious question. A larger and sampled scene set would allow the item-level variation we observed—effects concentrated on the longer, denser passages—to be modeled rather than merely noted. And because the response format proved decisive at three separate points in this study, the most transferable methodological lesson is that a spatial evaluation should vary its readout as deliberately as it varies its stimuli. Multimodal models that receive both text and a blank visual field, or an empty workspace in which an artificial gaze can be recorded, would allow the construct to move closer to the sensorimotor setting in which the human phenomenon was originally observed (Rayner, 2009; Spivey & Geng, 2001). Until such bridges are in place, the conclusion the present data support is a narrow one: current language models recover the spatial configuration of described scenes robustly, and without help from directional words, while the properties that appeared to limit that recovery belong largely to the instruments we used to measure it.

## 6. Conclusions

We asked whether the responses of current language models to spatial descriptions are governed by the configuration those descriptions convey or by the directional vocabulary they contain. Holding the described layout constant while varying its linguistic support, and reading the same layout out through a forced-choice direction report, an unconstrained narration, a two-dimensional reconstruction, and a plain relational question, separates the two more cleanly than a single direction question could. One result is stable across every variation we tested. Passages that convey a layout without naming a direction are answered correctly at near-ceiling rates, their objects are reconstructed in the right order, they are narrated in the right order, and the relations among their objects are reported correctly. This holds when the sentences are reordered, when the object list supplied in the prompt is shuffled, when the imagery framing is removed from the question, and when the models are given the option of declining to name a direction. Directional vocabulary is not what these responses are tracking.

The other result is a caution about method, and it applies as much to a single-format reading of these data as to the field. Three phenomena that would otherwise be taken as properties of the models proved to be properties of how they were measured. A misleading directional word appeared to be resisted three quarters of the time, but the same sentence moved from the start of the passage to its end is followed without a single exception, so what the manipulation measured was recency rather than the strength of a spatial commitment. Scenes without a dominant axis appeared to elicit a systematic downward bias, but the bias vanishes once declining is permitted, so it was an artifact of forcing a choice among four inapplicable options. Unconstrained narrations appeared unfaithful to the layout, but they are faithful to it when scored by the order in which they visit the objects, and the apparent deficit came from our imposing one direction on descriptions of wandering scanpaths. Each correction moved a finding out of the model and into the instrument.

We therefore end with a claim narrower than the one we set out to make, and with more confidence in it. These models recover the spatial configuration of a described scene from configuration alone, robustly and across two families and two generations. Whether anything in them resembles a spatial image is a question this design cannot reach and we do not raise. What the design does show is that separating layout from lexicon is both possible and necessary, and that spatial evaluations which vary only one response format will mistake the shape of their instrument for the shape of the ability they mean to measure.

## Figures and Tables

**Table 1:** Hypotheses, predicted effects, adjudicating contrasts, and decision criteria. Confirmatory contrasts were specified in advance of the analyses; exploratory contrasts are reported for completeness and are not used to support conclusions.

	Prediction	Adjudicating Contrast and Criterion	Status
H1	Responses track the described configuration, not directional vocabulary. Removing the directional word, and reordering the sentences that convey the layout, should leave accuracy near its ceiling.	Absolute accuracy on the implicit passages, which name no direction, together with the implicit-versus-scrambled contrast in the forced-choice task. Supported if implicit accuracy is near ceiling and the contrast does not reach significance across the forty cells after correction for multiple comparisons.	Confirmatory
H2	That governance is not absolute. A directional word asserting the opposite layout should lower accuracy.	Counter-cue versus congruent insertion in the forced-choice task, which holds the act of insertion constant. Supported if accuracy is lower under the counter-cue.	Confirmatory
H3	A categorical direction report and a relational reconstruction are dissociable. A counter-cue should perturb the former more than the latter, and scrambling should do the reverse.	The cue-by-task interaction pattern, tested as separate paired contrasts within each task. Supported if the ordering of conditions differs between tasks.	Confirmatory
H4	Verbal direction reports carry response biases that are not properties of the described scene.	Distribution of responses on non-directional control scenes, with and without an explicit indeterminate option. Supported if the distribution departs from uniformity and shifts when the option is offered.	Exploratory

**Table 2:** Spatial families in the stimulus set. Each directional family contributes one scene adapted from Spivey and Geng and one novel scene; control families have no dominant axis. Objects are listed in their correct spatial order along the dominant axis.

Direction	Source	Scene	Salient Objects in Spatial Order
Upward	Spivey	Apartment building	doorman, woman, smokers, screamers
Upward	Novel	Rocket launch	pad, tower, cloud, sky
Downward	Spivey	Canyon rappel	rim, first stop, second stop, floor
Downward	Novel	Old well	rim, moss, water mark, bottom
Leftward	Spivey	Standing train	engine, cargo car, flat car, caboose
Leftward	Novel	Mantel shelf	clock, vase, books, lamp
Rightward	Spivey	Fishing boat	fisherman, rod tip, line end, water
Rightward	Novel	Corridor	door, painting, window, exit
Control	Spivey	City telescope	block, building, window, family
Control	Novel	Desktop	cup, notebook, pen, phone

**Table 3:** Forced-choice direction accuracy by model and cue version, pooled over the eight directional scenes. Chance is 0.25 for the four-alternative choice.

Model	Explicit	Implicit	Scrambled	Conflict
Qwen3.7-max	1.000	0.963	1.000	0.744
Qwen3.7-plus	1.000	0.919	0.975	0.800
Qwen3.6-max-preview	1.000	0.956	0.994	0.781
Qwen3.6-plus	1.000	0.938	0.988	0.769
GLM-5.2	1.000	0.988	0.913	0.681
Overall	1.000	0.953	0.974	0.755

*Note*. Each model-by-cue cell aggregates 160 responses (eight scenes × twenty samples); the overall row aggregates 800 responses per cue version.

**Table 4:** Mean Kendall’s τ between the object ordering implied by reconstructed coordinates and the ground-truth ordering along the dominant axis, by model and cue version.

Model	Explicit	Implicit	Scrambled	Conflict
Qwen3.7-max	0.892	0.979	0.821	0.954
Qwen3.7-plus	0.996	0.996	0.617	0.925
Qwen3.6-max-preview	0.979	0.988	0.846	0.992
Qwen3.6-plus	1.000	0.992	0.662	0.971
GLM-5.2	1.000	1.000	0.629	0.875
Overall	0.973	0.991	0.715	0.943

*Note*. Each model-by-cue cell aggregates 80 reconstructions (eight scenes × ten samples). Control scenes were not administered the reconstruction task because their objects have no dominant spatial order. The values are for the original prompt, in which the object list was given in spatial order. The shuffled-list replication reported in the text is the measure of record; it does not differ reliably from these values in any condition.

**Table 5:** Outcomes in the counter-cue (conflict) condition by model. A response resists when it matches the true spatial layout and follows the word when it matches the inserted opposite direction. These figures are for the original sentence-initial placement of the misleading cue; as Section 4.3 shows, the same sentence placed after the layout is followed on every trial, so the resistance rates below are specific to cue position and are not a general property of the models.

Model	Resisted	Followed Word	Resistance Rate	Follow-Word Rate
Qwen3.7-max	119	41	0.744	0.256
Qwen3.7-plus	128	32	0.800	0.200
Qwen3.6-max-preview	125	35	0.781	0.219
Qwen3.6-plus	123	37	0.769	0.231
GLM-5.2	109	51	0.681	0.319
Overall	604	196	0.755	0.245

*Note*. Counts are out of 160 counter-cue responses per model (800 overall). No response fell into a residual category matching neither the layout nor the word.

## Data Availability

All materials, data, and code needed to reproduce the reported results are openly available at https://github.com/Foxbabe1q/Layout-over-Lexicon-Do-Large-Language-Models-Recover-Spatial-Structure-from-Scene-Descriptions- (accessed on 22 September 2026). The repository contains the complete stimulus set for every cue version and control condition, the verbatim prompt templates for all response formats, the unmodified raw responses returned by each model as newline-delimited JSON, the scored response table, and the scoring, statistical-analysis, and figure-generation scripts. Because responses obtained from a hosted API cannot be regenerated identically once a model version is retired, the raw responses rather than only the derived summaries are deposited.

## Abbreviations

The following abbreviations are used in this manuscript:

LLMs	Large language models
API	Application programming interface

## Appendix A. Stimulus Materials

Each directional scene was written in four cue versions that hold the described spatial layout constant while varying its linguistic support; the two control scenes exist in a single version. For each directional family the explicit version states the gaze direction outright, the implicit version conveys only the layout, the scrambled version reorders the implicit sentences without altering them, and the counter-cue version prepends a single sentence naming the opposite direction. The text of every scene in each of these four cue versions is listed in Table A1, Table A2, Table A3, Table A4 and Table A5 grouped by the dominant direction of the scene. The additional control materials are given separately in Table A6: because each of the four additional insertion conditions is the implicit passage plus exactly one sentence, only the inserted sentences need be listed, and the four additional control scenes are given in full. Every passage is reproduced exactly as it was presented to the models, including the original orthography of the sentences taken from Spivey and Geng, so that the materials in this appendix are the strings that were actually sent to the API.

**Table A1:** Upward scenes in all cue versions.

Version	Text
Apartment (Spivey)	
Explicit	Imagine standing across the street from a 40-story apartment building; your gaze travels upward. At the bottom is a doorman in blue; higher up on the 10th floor a woman hangs her laundry; higher still on the 29th floor two kids smoke on the fire escape; and at the very top two people are screaming.
Implicit	Imagine that you are standing across the street from a 40 story apartment building. At the bottom there is a doorman in blue. On the 10th floor, a woman is hanging her laundry out the window. On the 29th floor, two kids are sitting on the fire escape smoking cigarettes. On the very top floor, two people are screaming.
Scrambled	Imagine that you are standing across the street from a 40 story apartment building. On the 29th floor, two kids are sitting on the fire escape smoking cigarettes. At the bottom there is a doorman in blue. On the very top floor, two people are screaming. On the 10th floor, a woman is hanging her laundry out the window.
Counter-cue	As you take it in, your gaze drifts downward. Imagine that you are standing across the street from a 40 story apartment building. At the bottom there is a doorman in blue. On the 10th floor, a woman is hanging her laundry out the window. On the 29th floor, two kids are sitting on the fire escape smoking cigarettes. On the very top floor, two people are screaming.
Rocket (novel)	
Explicit	Imagine watching a rocket launch. It ignites on the ground and climbs upward, first rising past the launch tower, then higher through the clouds, and finally vanishing far above in the sky.
Implicit	Imagine you are watching a rocket at a launch site. It ignites on the launch pad at ground level. It clears the top of the launch tower. It disappears into a thick layer of cloud. It becomes a tiny bright dot in the far sky.
Scrambled	Imagine you are watching a rocket at a launch site. It disappears into a thick layer of cloud. It ignites on the launch pad at ground level. It becomes a tiny bright dot in the far sky. It clears the top of the launch tower.
Counter-cue	As you take it in, your gaze drifts downward. Imagine you are watching a rocket at a launch site. It ignites on the launch pad at ground level. It clears the top of the launch tower. It disappears into a thick layer of cloud. It becomes a tiny bright dot in the far sky.

**Table A2:** Downward scenes in all cue versions.

Version	Text
Canyon rappel (Spivey)	
Explicit	Imagine standing at the top of a canyon, watching a person rappel down the far wall. From the rim she drops downward, is caught, then descends lower and lower in successive jumps until she reaches the floor.
Implicit	Imagine you are standing at the top of a canyon. Several people are preparing to rappel down the far canyon wall across from you. The first person descends 10 feet before she is brought back to the wall. She jumps again and falls 12 feet. She jumps another 15 feet. And the last jump, of 8 feet, takes her to the canyon floor.
Scrambled	Imagine you are standing at the top of a canyon. She jumps another 15 feet. Several people are preparing to rappel down the far canyon wall across from you. And the last jump, of 8 feet, takes her to the canyon floor. The first person descends 10 feet before she is brought back to the wall. She jumps again and falls 12 feet.
Counter-cue	As you take it in, your gaze drifts upward. Imagine you are standing at the top of a canyon. Several people are preparing to rappel down the far canyon wall across from you. The first person descends 10 feet before she is brought back to the wall. She jumps again and falls 12 feet. She jumps another 15 feet. And the last jump, of 8 feet, takes her to the canyon floor.
Old well (novel)	
Explicit	Imagine leaning over the mouth of an old well and looking down. Your gaze drops lower and lower: the stone rim, then the mossy shaft below it, then a faint water mark further down, and finally the dark bottom.
Implicit	Imagine leaning over the mouth of an old well and looking in. The rim is a ring of polished stone. Beyond it is a shaft covered in moss. Then comes a faint old water mark. At the end is the dark, unseeable bottom.
Scrambled	Imagine leaning over the mouth of an old well and looking in. Then comes a faint old water mark. The rim is a ring of polished stone. At the end is the dark, unseeable bottom. Beyond it is a shaft covered in moss.
Counter-cue	As you take it in, your gaze drifts upward. Imagine leaning over the mouth of an old well and looking in. The rim is a ring of polished stone. Beyond it is a shaft covered in moss. Then comes a faint old water mark. At the end is the dark, unseeable bottom.

**Table A3:** Leftward scenes in all cue versions.

Version	Text
Standing train (Spivey)	
Explicit	Imagine a train extending to the left. You face the side of the engine, which points right. Moving your gaze leftward: a cargo car with pink graffiti, then further left a flat car, and at the far left the caboose coming around a corner.
Implicit	Imagine a train extending outwards to the left. It is pointed to the right, and you are facing the side of the engine. It is not moving. Five cars down is a cargo holder with pink graffiti sprayed on its side. Another six cars down is a flat car. The train begins to move. Further down the train you see the caboose coming around a corner.
Scrambled	Imagine a train extending outwards to the left. Another six cars down is a flat car. It is pointed to the right, and you are facing the side of the engine. The train begins to move. It is not moving. Further down the train you see the caboose coming around a corner. Five cars down is a cargo holder with pink graffiti sprayed on its side.
Counter-cue	As you take it in, your gaze drifts to the right. Imagine a train extending outwards to the left. It is pointed to the right, and you are facing the side of the engine. It is not moving. Five cars down is a cargo holder with pink graffiti sprayed on its side. Another six cars down is a flat car. The train begins to move. Further down the train you see the caboose coming around a corner.
Mantel shelf (novel)	
Explicit	Imagine a long shelf; your gaze sweeps leftward from the right end. At the far right a mantel clock, then to the left a vase, further left a stack of books, and at the far left a lamp.
Implicit	Imagine a long shelf; you are standing at its right end. By your right hand is a mantel clock. A space away is a porcelain vase. Another space away is a stack of old books. At the end of the shelf sits a lamp.
Scrambled	Imagine a long shelf; you are standing at its right end. Another space away is a stack of old books. By your right hand is a mantel clock. At the end of the shelf sits a lamp. A space away is a porcelain vase.
Counter-cue	As you take it in, your gaze drifts to the right. Imagine a long shelf; you are standing at its right end. By your right hand is a mantel clock. A space away is a porcelain vase. Another space away is a stack of old books. At the end of the shelf sits a lamp.

**Table A4:** Rightward scenes in all cue versions.

Version	Text
Fishing boat (Spivey)	
Explicit	Imagine a fishing boat pointing left. At the stern a fisherman holds a rod; the rod extends rightward past the hull, and from its tip the line runs far to the right before dipping into the water.
Implicit	Imagine a fishing boat floating on the ocean. It’s facing leftward from your perspective. At the back of the boat is a fisherman with a fishing pole. The pole extends about 10 feet to the right beyond the edge of the boat. From the end of the pole, the fishing line extends another 50 feet before finally dipping into the water.
Scrambled	Imagine a fishing boat floating on the ocean. The pole extends about 10 feet to the right beyond the edge of the boat. It’s facing leftward from your perspective. From the end of the pole, the fishing line extends another 50 feet before finally dipping into the water. At the back of the boat is a fisherman with a fishing pole.
Counter-cue	As you take it in, your gaze drifts to the left. Imagine a fishing boat floating on the ocean. It’s facing leftward from your perspective. At the back of the boat is a fisherman with a fishing pole. The pole extends about 10 feet to the right beyond the edge of the boat. From the end of the pole, the fishing line extends another 50 feet before finally dipping into the water.
Corridor (novel)	
Explicit	Imagine standing at the left end of a corridor, looking rightward. Nearest is a wooden door, then to the right a painting, further right a window, and at the far right an exit to the outside.
Implicit	Imagine standing at the left end of a long corridor. Beside you is a wooden door. A stretch along the wall hangs a painting. Farther along the wall is a window. At the end of the corridor is an exit to the outside.
Scrambled	Imagine standing at the left end of a long corridor. Farther along the wall is a window. Beside you is a wooden door. At the end of the corridor is an exit to the outside. A stretch along the wall hangs a painting.
Counter-cue	As you take it in, your gaze drifts to the left. Imagine standing at the left end of a long corridor. Beside you is a wooden door. A stretch along the wall hangs a painting. Farther along the wall is a window. At the end of the corridor is an exit to the outside.

**Table A5:** Non-directional control scenes (single version each).

Scene	Text
City telescope (Spivey)	Imagine you are on a hill looking at a city through a telescope. One button zooms a specific block into view; another brings a gray apartment building into focus; a third zooms in on a single window, where a family is having breakfast together.
Desktop (novel)	Imagine a desk. On it are a coffee cup, a notebook, a pen, and a phone, casually arranged together.

**Table A6:** Additional control materials. Each insertion condition is the implicit passage of Table A1, Table A2, Table A3 and Table A4 with exactly one sentence added and nothing else altered, so only the inserted sentences are listed; the sentence is prepended except in the final-position condition, where it is appended. The direction named is the true direction of the scene in the congruent condition and its opposite in the other three. The four control scenes are given in full and were presented in a single version.

Condition	Inserted Sentence, by the Direction It Names
Insertion conditions (prepended unless stated)	
Congruent	As you take it in, your gaze drifts upward./downward./to the left./to the right.
Final position	The same four sentences as the counter-cue condition, appended after the layout instead of before it.
Reworded	Note that the overall movement here runs upward./downward./leftward./rightward.
Non-spatial	As you take it in, you recall that rents in this neighbourhood have been drifting upward (downward) for years./As you take it in, you recall that local politics had been drifting leftward (rightward) for years.
Additional non-directional control scenes	
Round table	Imagine a small round table set for a meal. A plate, a glass, a folded napkin, and a short candle are placed around it at even spacing.
Toolbox	Imagine an open toolbox. Inside it a hammer, a screwdriver, a pair of pliers, and a roll of tape lie jumbled together in no particular order.
Backpack	Imagine a backpack that has just been unzipped. Packed against one another inside are a laptop, a water bottle, a notebook, and a pair of headphones.
Noticeboard	Imagine a cork noticeboard. Pinned to it are a calendar, a postcard, a receipt, and a photograph, overlapping one another at various angles.

## Appendix B. Prompts and Representative Responses

Each of the thirty-four core stimulus items was probed with the forced-choice and free-form prompts below and each of the thirty-two directional items with the reconstruction prompt as well. In each case the scene text from Appendix A was appended to the instruction.

*Forced-choice direction.* You are in an imagination experiment. Read the scene below, imagine it with your eyes closed, and report the main direction your gaze moves. Choose exactly one of [up, down, left, right] and give a one-sentence reason. Answer strictly in this format, nothing else: Direction: X; Reason: …

*Free-form gaze.* Read the scene below, imagine it, and naturally describe how your gaze moves as you imagine it, and why.

*Reconstruction.* Read the scene below and place its main objects on a 2D grid. The x-axis is positive to the right, negative to the left; the y-axis is positive up, negative down. Output one line per object in the form ‘object: (x, y)’ with integer coordinates, nothing else. Objects: doorman, woman, smokers, screamers.

Representative responses to the upward apartment scene (Qwen3.6-max-preview) illustrate the three answer formats.

*Forced-choice direction.* Direction: up; Reason: The narrative sequentially directs attention from the bottom of the building to progressively higher floors, culminating at the very top.

*Free-form gaze.* My gaze begins at eye level, anchored by the doorman in blue at the base of the building …From there, my eyes naturally drift upward, following the vertical lines of the architecture …I pause briefly at the 10th floor, where the woman hangs laundry, before continuing toward the top.

*Reconstruction.* doorman: (0, 1); woman: (0, 10); smokers: (0, 29); screamers: (0, 40).

The counter-cue condition is resolved as a choice between the true layout and the inserted word. For the apartment scene, whose true direction is upward but whose prepended sentence names “downward,” both outcomes occur across repeated samples.

*Resisting the word (true layout).* Direction: up; Reason: Although the scene begins with a downward drift, the narrative structure progressively lists details from the bottom to the top of the building, culminating in the action on the highest floor which naturally draws the gaze upward.

*Following the word.* Direction: down; Reason: The scene explicitly states that your gaze drifts downward.

The four additional response formats are given below. The shuffled reconstruction differs from the original only in the parenthetical disclaiming the list order; the neutral direction prompt removes every reference to imagination and gaze while leaving the alternatives and answer format untouched; the indeterminate prompt is the original forced-choice prompt with a fifth alternative; and the relational prompt asks about the described scene rather than about the model’s viewing behavior, substituting the first- and last-introduced objects of the scene.

*Reconstruction, shuffled object list.* Read the scene below and place its main objects on a 2D grid. The x-axis is positive to the right, negative to the left; the y-axis is positive up, negative down. Output one line per object in the form ‘object: (x, y)’ with integer coordinates, nothing else. Objects (listed in no particular order): smokers, screamers, doorman, woman.

*Direction, neutral framing.* Read the description below and report the main direction along which the described scene extends. Choose exactly one of [up, down, left, right] and give a one-sentence reason. Answer strictly in this format, nothing else: Direction: X; Reason: …

*Direction, indeterminate option.* You are in an imagination experiment. Read the scene below, imagine it with your eyes closed, and report the main direction your gaze moves. Choose exactly one of [up, down, left, right, none], where none means the scene has no single dominant direction. Give a one-sentence reason. Answer strictly in this format, nothing else: Direction: X; Reason: …

*Spatial relation.* Read the description below and report a spatial relation between two of the things it mentions: where is the screamers relative to the doorman? Choose exactly one of [above, below, left, right] and give a one-sentence reason. Answer strictly in this format, nothing else: Relation: X; Reason: …
